# Disease Modifying Strategies in Multiple Sclerosis: New Rays of Hope to Combat Disability?

**DOI:** 10.2174/1570159X22666240124114126

**Published:** 2024-01-24

**Authors:** Carlo Maria Bellanca, Egle Augello, Alice Mariottini, Gabriele Bonaventura, Valentina La Cognata, Giulia Di Benedetto, Anna Flavia Cantone, Giuseppe Attaguile, Rosaria Di Mauro, Giuseppina Cantarella, Luca Massacesi, Renato Bernardini

**Affiliations:** 1 Department of Biomedical and Biotechnological Sciences (BIOMETEC), Section of Pharmacology, University of Catania, 95123 Catania, Italy;; 2 Clinical Toxicology Unit, University Hospital, University of Catania, 95123 Catania, Italy;; 3 Department of Neurosciences Drugs and Child Health, University of Florence, Florence, Italy;; 4 Institute for Biomedical Research and Innovation (IRIB), Italian National Research Council, 95126 Catania, Italy

**Keywords:** Multiple sclerosis, disease-modifying therapies, clinical trials, approved drugs, off-label treatments, future perspective, stem cells

## Abstract

Multiple sclerosis (MS) is the most prevalent chronic autoimmune inflammatory- demyelinating disorder of the central nervous system (CNS). It usually begins in young adulthood, mainly between the second and fourth decades of life. Usually, the clinical course is characterized by the involvement of multiple CNS functional systems and by different, often overlapping phenotypes. In the last decades, remarkable results have been achieved in the treatment of MS, particularly in the relapsing-remitting (RRMS) form, thus improving the long-term outcome for many patients. As deeper knowledge of MS pathogenesis and respective molecular targets keeps growing, nowadays, several lines of disease-modifying treatments (DMT) are available, an impressive change compared to the relative poverty of options available in the past. Current MS management by DMTs is aimed at reducing relapse frequency, ameliorating symptoms, and preventing clinical disability and progression. Notwithstanding the relevant increase in pharmacological options for the management of RRMS, research is now increasingly pointing to identify new molecules with high efficacy, particularly in progressive forms. Hence, future efforts should be concentrated on achieving a more extensive, if not exhaustive, understanding of the pathogenetic mechanisms underlying this phase of the disease in order to characterize novel molecules for therapeutic intervention. The purpose of this review is to provide a compact overview of the numerous currently approved treatments and future innovative approaches, including neuroprotective treatments as anti-LINGO-1 monoclonal antibody and cell therapies, for effective and safe management of MS, potentially leading to a cure for this disease.

## 1 INTRODUCTION

Multiple sclerosis (MS) is a chronic autoimmune inflammatory and demyelinating disorder affecting the central nervous system (CNS) with potentially devastating and long-term complications, resulting in progressive neurodegeneration and neurological disability [1]. Although the data from underdeveloped countries may not be accurate, recent reports show that in 2020, MS affected more than 2.8 million people worldwide, with a global median prevalence of 36 cases per 100,000 people and a relevant variance between countries. Where the data are reliable, North America and Europe are the countries with the highest prevalence (with 300 and 250 per 100,000 people, respectively), and Asia as well as sub-Saharan Africa have the lowest prevalence (2.2 and 2.1 per 100,000 people, respectively) [2-4].

MS primarily affects young adults (more commonly women), with an age of onset mainly between 20 and 40 years [5, 6]. Compared with the general population, MS patients have a higher mortality rate and shorter lifetime expectancy, especially those with comorbidities such as psychiatric disorders, cerebrovascular and cardiovascular diseases, diabetes, or cancer [7]. Although MS is not considered a life-threatening terminal illness, it still cannot be cured as most therapies usually just modify disability trajectories but leave patients with a reduced quality of life for extended periods of time.

The aetiology of the disease is still uncertain, but the most updated working models for disease pathogenesis propose an interplay between genetic and environmental factors as necessary for MS manifestation [8].

The strongest genetic association signal in MS resides within the major histocompatibility complex (MHC) in chromosome 6p21.3. This 4-megabase region contains approximately 160 closely linked genes. About half of these genes have important roles in the regulation of the immune system and include the six classical transplantation human leukocyte antigen (HLA) genes, the class I genes HLA-A, HLA-B, and HLA-C, and the class II genes HLA-DPB1, HLA-DQB1, and HLA-DRB1 [1]. During the past decade, the introduction of genome-wide association studies (GWAS) with increasing sample size has revolutionized the genetics of MS, determining the discovery of a wide spectrum of robustly associated genetic variants at an exponential rate. To date, more than 200 genetic loci have been uncovered that independently contribute to disease pathogenesis, mainly involved in the immune system and related mechanisms [8].

Along with the genetic background, MS also arises from environmental factors, including Epstein-Barr virus (EBV) infection, tobacco smoking, obesity, diet, air pollution, and radiation exposure [9, 10]. Interestingly, MS prevalence increases with latitude and is strongly inversely correlated to UVB exposure, which stimulates cutaneous vitamin D (vD) production. The vD implication in the causal pathway of MS is related to low levels, decreased intake, reduced outdoor activity, and genetic polymorphisms causing low vD levels [11].

Despite the advancements in our knowledge of MS, many challenges and unknowns remain to be explored, given the risk for this disease driven by multiple common variants whose biological effects are still not completely explained [8, 12].

## 2 METHODS

A search of the relevant literature (up to January 2023) was conducted on MEDLINE (PubMed), ClinicalTrial.gov, and Google Scholar, applying the medical subject headings (MeSH) terms “multiple sclerosis”, “approved therapeutic approaches”, “off-label therapeutic approaches”, “disease-modifying treatments”, “emerging therapies”, “clinical trials”, “observational studies”, “escalation treatment”, early active treatment”, “high-efficacy therapies”, “stem-cell based approaches”, “safety”, and “efficacy”. From the web-based search, we selected peer-reviewed, full-text, and English-language manuscripts. Randomized controlled trials (RCTs) with their extension trials and sub-studies, prospective studies, non-randomized clinical trials, observational studies, and reviews were included. We excluded single case studies, paediatric studies, and non-peer-reviewed publications. Each selected paper was preliminarily examined by both senior authors (*via* abstract reading), downloaded, and summarized.

## 3 INFLAMMATORY DEMYELINATING MECHANISMS

In MS patients, the progressive demyelination and diffuse degeneration of nerve fibres and neurons in the white and grey matter of the brain and spinal cord is secondary to an immune response occurring in the CNS promoted by adaptative immunity. Effector antigen (Ag-)specific lymphocytes, indeed, infiltrate the CNS, initially in the perivenular spaces, from where inflammatory cytokines and other damaging factors are released in the surrounding tissue, leading to a cascade of sequential events including blood-brain barrier (BBB) disruption, demyelination and eventually axon damage [13-16]. The consequence is usually an initially remitting tissue damage and dysfunction of the corresponding functional system. However, over time, lesion accumulation makes both the tissue and the function disruption irreversible [17]. In most patients, indeed, the disease starts with a relapsing-remitting course (RRMS), which is followed after several years by a secondary progressive phase (SPMS) [18]. Most patients, thus, - usually between 5 to 20 years from the diagnosis - develop an insidious, progressive disease course characterized by high lesion load, low rate of new lesion accumulation, compartmentalization of the chronic CNS lesion, and persistent or progressive clinical disability due to persistent axonal loss. Anti-inflammatory or immunosuppressive drugs, such as anti-CD20 drugs (*e.g*., rituximab, ocrelizumab, and ofatumumab), and anti-CD52 drugs (alemtuzumab) involving B and T-lymphocytes, are indeed beneficial in patients with MS with an efficacy inversely proportional to disease duration, being therefore high early in the course of the RRMS form, but low or absent in progressive disease [19]. Current therapies often cannot prevent the accumulation of permanent disability due to axonal and neuronal damage and loss [20]. This limitation has led to the search for a new strategy to be added to the currently available ones, *i.e*., protect against neurodegeneration to avoid progressive disability. However, this strategy, as in other neurodegenerative diseases, such as motor neuron disease, stroke, and Alzheimer’s disease, has been thus far difficult to pursue.

However, as in MS, the primary event is demyelination; restoring myelin (remyelination or myelin regeneration) seems a more feasible therapeutic strategy that, beyond restoring or improving nerve conduction *per se,* determines nerve fibres protection, providing mechanical as well metabolic support to the underlying axon [18]. Besides, this approach could be used as a therapeutic test bed for other neurodegenerative conditions for which white matter abnormalities and demyelination have been identified [21].

Myelin is a vertebrate-specific structure consisting of membrane wrapped spirally around large-diameter axons forming sheaths with a unique biochemical constitution consisting of 70% lipids and 30% proteins, the opposite of other membranes. In the CNS, myelin sheaths are generated by myelinating oligodendrocytes (OLs), differentiated and maturated from oligodendrocyte precursor cells (OPCs and by Schwann cells in the peripheral nervous system) [22]. This process also persists in adult life, the reason by which myelin is one of the few CNS structures capable of regenerating after damage.

Between myelin sheaths, Ranvier nodes can be observed at regular intervals. At these nodes, sodium channels are clustered, and the action potential jumps from one node to the next, allowing rapid transmission of the action potential along the axons, known as saltatory conduction (50-100 times faster than along unmyelinated axons of similar diameter). In non-myelinated fibres indeed, voltage-dependent sodium channels are evenly distributed along the axons, causing the nerve impulse to move continuously. Besides their effect on conduction, through the myelin sheaths, oligodendrocytes also provide mechanical and metabolic support to the underlying axons, fuelling axonal mitochondria and crucially supporting energy-demanding axonal transport. Potassium clearance, through oligodendrocyte-specific potassium ion channels, is also crucial for axonal function and integrity [23, 24].

In MS, the direct effect of demyelination is a lack of axon support with indirect neural damage and loss. A lot of indirect mechanisms, indeed, contribute to the collateral axon damage caused by demyelination [25]. These mechanisms include the redistribution of voltage-dependent sodium channels along the denuded axon, the altered clearance of potassium ions due to the lack of oligodendrocytes potassium channels and finally, the lack of nutritional support, which leads to an increase in the energy demand of the axon [26, 27].

However, it is currently unknown whether, in specific MS forms, even axons and/or neurons may represent a primary target of the autoimmune attack, a hypothesis that could explain the more disabling course of this disease usually not associated with aggressive inflammation, as seen in PPMS.

For more detailed information, we send the reader to paragraphs describing each single drug.

## 4 CLINICAL FEATURES AND DIAGNOSIS

During the acute phases of MS, symptoms and signs vary according to both the location of the CNS region affected by the underlying lesions and the severity/size of the associated inflammatory process. Symptoms may, therefore, result from the involvement of any functional systems (*i.e*., motor, sensory, visual) proportionally affected according to their extension.

As a result of the increased need for harmonized terminology, in 1996, the *U.S. National Multiple Sclerosis Society* (NMSS) *Advisory Committee on Clinical Trials in Multiple Sclerosis* introduced the first formally defined MS phenotypes, conscious that they would need to be implemented, owing to their purely clinical nature. The terms relapsing-remitting MS (RRMS), primary progressive MS (PPMS), secondary progressive MS (SPMS), and progressive relapsing MS (PRMS) were proposed, with the purpose of ensuring the proper design of clinical trials and the homogeneity of patients recruited, providing a strong foundation for successful outcomes [28].

Despite their theoretical usefulness, the aforementioned phenotypes often failed to reflect the clinical reality, and attempts were made to further refine definitions of the clinical MS forms. Particularly when MRI biological markers and other assays, including neurophysiology, become available, the need to incorporate them as tools that could provide objective criteria for distinguishing phenotypes, in 2013 prompted the re-examination of MS clinical subtypes by the Committee [29].

Clinically isolated syndrome (CIS), originally not included in MS clinical descriptors, was then recognized as an established disease course. The term CIS identifies the first clinical event consistent with a demyelinating CNS syndrome that might be suggestive of MS (including DIT) but has not yet met MRI criteria for dissemination in time (DIT). The vast majority of patients experience a single episode due to inflammatory demyelination, which involves the brain hemispheres, optic nerve, brainstem, or spinal cord, evolves acutely or sub-acutely over days to weeks, and resolves in the course of time [30].

The term radiologically isolated syndrome (RIS), first introduced by Okuda in 2009 [31], was adopted to indicate patients showing MRI abnormalities who are likely to develop a clinically definite MS [32]. The wide application of MRI in clinical practice has dramatically increased the incidental discovery of white matter lesions highly suggestive of demyelination in patients with no clinical evidence of any disease. To date, few studies have explored the potential clinical evolution of radiologically isolated syndrome (RIS) patients. Therefore, further investigations with the aim of assessing baseline factors with prognostic relevance for either clinical or radiological progression are of high importance [32].

Approximately 85-95% of MS patients initially follow an RR clinical course, defined by an alternation of acute exacerbations (relapses), followed by complete or incomplete remissions and periods of relative clinical stability in the meantime. An exacerbation is defined as a patient-reported or objectively observed by the examining neurologist new or recurrent neurological manifestation, lasting at least 24 hours in the absence of fever or infection [33].

In untreated patients, the transition from RRMS to SPMS is expected to take place after about 5-20 years [34, 35]. In the past, up to 40% of patients eventually experienced an accrual of fixed disability and a gradual worsening of neurological functioning, often mostly involving CNS areas most severely affected during the relapsing-remitting course, as motor and spinocerebellar systems or cognitive functions [36]. Even if few are known for the long-term outcomes following the recent introduction of highly active treatments, a remarkable reduction of long-term disability accrual is expected (see below).

Finally, the PPMS phenotype identifies patients with progressive neurological function deterioration from the outset.

To adequately capture the complexity of MS phenotypes, additional modifiers were added to the original categorizations. Each single subtype of disease should be accompanied by the terms “active” or “not active”, aiming to add information about disease activity status and “progressive” if clinical or instrumental evidence of progression is present [37].

More recently, the dichotomic classification into RRMS and SPMS was challenged by the observation that many patients gradually switch from the RR to the SP phenotype, as accrual of disability independent of new overt inflammation may be observed in RRMS. This phenomenon was first described as “silent progression” [38], and then further characterized as Progression Independent from Relapse Activity (PIRA) [35]. The occurrence of PIRA since early RRMS [39] suggests that inflammatory and degenerative pathogenetic mechanisms may be established since MS onset, arguing that MS progresses along a continuum from relapsing to progressive disease [40].

PIRA is pathologically based on two chronic lesion types: inflammatory active lesions and non-inflammatory active, purely degenerative scars.

The inflammatory active lesions evolve from previous new lesions, containing adaptative immunological processes persisting in the perivenular infiltrates and innate immunological processes evolving at their edges; both compartmentalized within the CNS behind a healed BBB. These lesions, first described in Experimental Autoimmune Encephalitis (EAE) and later in MS, and now widely known as *smouldering* lesions, increase in terms of size and number during the course of the disease, being more frequent in patients experiencing PIRA and in the SP phenotype and being associated to high disability and disability progression rate [41-45]. The development of chronic infiltrates in MS also includes a progressive accumulation of chronic, meningeal inflammatory follicles resembling tertiary lymphoid tissue, at least in part compartmentalized [46], whose presence can, therefore, be estimated by the only presence of cortical lesions abutting on the meninges [47]. However, some of them, probably the new ones, can be visualized by contrast MRI, indicating that they are building around vessels with damaged or incomplete BBBs [46, 48].

The non-inflammatory active, purely degenerative scars probably develop from the inflammatory lesions in which inflammation has eventually vanished and scarring processes have taken over. These lesions, which probably represent the only *plaques* fully deserving this denomination, are pathologically deeply different from the inflammatory active ones, as perivenular adaptive and perilesional innate immunity is no longer present, myelin and axon have been wiped away, and a hard grey gliotic scar made by astrocytes has been (or is being) developed [43]. However, *in vivo*, these lesions cannot be distinguished either by MRI, making it very difficult to prevent patients whose clinical progression is mainly due to these lesions from being treated with immunosuppressive drugs, as for them, the risk/benefit ratio is probably unfavourable.

### 4.1 DIAGNOSIS

The diagnosis is based on the integration of clinical, radiological, and laboratory findings with the aim to verify, in typical demyelinating syndromes, evidence of the dissemination in time (DIT) and space (DIS) criteria while excluding alternative diagnosis of a better explanation of the clinical picture, in cases meeting the diagnostic criteria of other neurological disorders with similar clinical manifestation [49]. DIS is defined as the development of lesions affecting at least two CNS areas typically involved in MS, whereas DIT refers to the requirement that symptoms have relapsed or CNS lesions have accumulated over time. MRI plays a crucial role in the early demonstration of DIS and DIT markers in the CNS. Prior to its availability, MS diagnosis was entirely based on clinical findings [36].

Nowadays, the most widely used diagnostic criteria for MS are the “McDonald’s” criteria, which were drawn up in 2001 [50] and revised in 2005 [51], 2010 [52], and 2017 [53]. They formally incorporated for the first time definitions of MRI criteria, thus allowing an earlier diagnosis and facilitating earlier treatment when appropriate [49]. Although the McDonald’s criteria are currently widely accepted, having improved precision and timing of the diagnosis thanks to the high sensitivity and good reproducibility, they recommend applying them only to patients presenting with typical symptoms of CIS or MS and in whom the disease is highly probable, and not to differentiate MS from other neurological disorders. Indeed, when clinical presentation is not typical for MS or when red flags of alternative diagnosis are present, these criteria should be applied with caution [54-56]. To our opinion, this means that in cases with non-typical syndromes or in those carrying red flags of alternative diagnosis (although without fulfilling diagnostic criteria of different diseases), MS diagnosis should be delayed, providing more certainty, at least until clinically established DIT occurs. In the meanwhile, a combination of history, clinical features, MRI, and serological testing should be used to navigate through the differential diagnosis of idiopathic inflammatory disorders and other conditions that can mimic MS until better explanations of the clinical presentation and alternative diagnosis are not definitively excluded. However, when differential diagnosis with alternative autoimmune-inflammatory disease involving the CNS cannot be excluded, we believe that nonspecific immunosuppressive medications (*i.e*., not developed just for MS) should be preferred [56].

A number of studies indeed demonstrated poor specificity of the McDonald’s diagnostic criteria, particularly when not applied in the proper setting [57] or when DIT is based on MRI findings only, as in the most recent iterations of the McDonald’s criteria [58]. Instead, the tension between this limitation of the most widely used diagnostic criteria and the pressure due to anticipation of the diagnosis for early treatment administration is determining a high MS misdiagnosis rate, to date estimated about 15% of cases, with subsequent inappropriate DMTs administration in patients carrying pathologies different from MS [55].

For these reasons, in the future, diagnostic criteria are going to be refined, possibly including new markers that can now be detected by MRI. Although still confined in a research setting, due to the need for not routinely available high-field MRI sequences and time-consuming post-processing analysis, increasing evidence indicates that, when widely applicable, new brain MRI markers will profoundly improve the accuracy of the MS diagnosis. In our opinion, indeed, this MRI marker will change the diagnostic approach to MS as it *in vivo* visualizes a pathological hallmark of MS, that is the perivenular location of the majority of the white matter demyelinating lesions, a characteristic that among the demyelinating MS-like syndromes is unique to MS [45, 59].

## 5 MEDICATIONS FOR MANAGEMENT OF MULTIPLE SCLEROSIS

Notable progress in the management of MS, particularly in the relapsing-remitting form, has modified the long-term outcome for many patients. Current management is supposed to reduce acute attack frequency, ameliorate symptoms, and prevent disability arising and progression *via* disease-modifying therapies (DMTs), which act on MS pathophysiological events through suppression or modulation of immune response. An aggressive approach involves the even more frequent adoption of “no evidence of disease activity” (NEDA) as a treatment target, defined by clinical and MRI criteria [60]. NEDA combines three related measures of disease activity: relapse absence, disability progression, and no MRI activity (new or enlarging T2-lesions and gadolinium (Gd)-enhancing lesions) [61].

In the next paragraphs, the current therapeutic strategies used to manage MS patients will be summarized. A timeline of US Food and Drug Administration (FDA) licensed drugs is available in Fig. (**1**). FDA data only were presented because the European Medicine Agency (EMA) has been activated only following the legislation released by the European Parliament in the year 2003. For the purpose of this review, it must be noted that according to this legislation, the evidence just proving efficacy and favourable risk/benefit ratio is considered sufficient for granting approval of a new treatment for a given indication. This means that should other treatments for the same indication be already approved, proof of superiority would not be required. As a consequence, the reader of this review and the European physicians must be aware that the more recent drugs approved by EMA may not necessarily be superior to the older ones unless direct head-to-head prospective comparisons have been included in their development. In addition, when the new medications, sponsored by the oversized commercial department of the marketing authorization holders, are approved, the patent of the old ones often expires, as well as their commercial support.

Despite several on-label DMTs holding marketing authorization for multiple sclerosis, as many off-label are also utilized in clinical practice. For instance, azathioprine, rituximab, methotrexate, cyclophosphamide, and mycophenolate mofetil, are among effective off-label options. They are registered medicinal products that, under given conditions, can be used for a purpose different than the authorised indications stated in the summary of product characteristics (SMPC). Although these molecules have been on the market for a long and are used in clinical conditions not considered by regulatory agencies, they still are used because of sufficient scientific evidence of efficacy. It is noteworthy that the quantity and quality of clinical evidence supporting the use of these off-label DMTs may vary significantly.

When it comes to off-label treatments, there is a need to offer clear guidance to clinicians, policymakers, and patients to optimize health outcomes. Upholding the utmost ethical principles involves ensuring equal access to an array of evidence-based therapies for all individuals. Employing off-label DMTs might be deemed unethical if more suitable and affordable on-label medications are accessible. On the other hand, refraining from using them could also be considered unethical when on-label alternatives are unavailable or financially burdensome, and off-label treatments prove to be both effective and safe [62].

The reasons behind off-label prescribing can be intricate, influenced by factors such as the local healthcare infrastructure and the availability of innovative drugs, generics, and biosimilars. In low-resource settings, off-label prescribing frequently stems from a shortage of suitable choices or cost considerations [63].

### 5.1 Azathioprine (AZA, Jayempi^®^)

For four decades, Azathioprine (AZA) has been used as an immunosuppressive and anti-inflammatory agent in organ transplantation (kidney and heart) [64, 65] and in chronic inflammatory diseases, including MS [66-73]. AZA is a prodrug selectively converted to the purine analogue 6-mercaptopurine in target cells, and purine nucleotide biosynthesis inhibition and downregulation of B and T cell function have been suggested as its main mechanism of action [74-78]. Furthermore, AZA (and its metabolites) can induce apoptosis of T cells through CD28 co-stimulation, mediated by a specific binding of azathioprine-generated 6-thioguanine triphosphate to Rac1 (Ras-related C3 botulinum toxin substrate 1) instead of guanosine-triphosphate, converting a costimulatory into an apoptotic signal. 6-Thio-GTP derivates, therefore, exert their immunosuppressive activity at least in part through slow but quite selective mechanisms [79].

In MS, AZA has been administered in many countries for at least four decades. This use is based on placebo-controlled randomized clinical trials (RCTs) [66, 80-82], although initially efficacy was considered marginal [83, 84], and after approval of β interferons (IFNs), AZA was no longer recommended as first-line therapy [85]. To our opinion, the inclusion of SPMS patients and lack of MRI assessment in the abovementioned RCTs (thus weakening their power), might have contributed to the perception of poor efficacy of AZA. Meta-analyses [86-88], new comparative RCTs [89, 90], and MRI results [91, 92] suggest a similar effect size of AZA in RRMS, and an independent multicenter RCT evaluating the non-inferiority of the efficacy of AZA *vs*. IFNs on clinical and MRI measures of disease activity (relapses and new brain lesions) in RRMS conclusively proved that its effect size is equivalent to that of the IFNs and, by extension, is equivalent to the curently available first line treatments for MS with effectiveness similar to IFNs. In addition, AZA seems also similarly efficacious to IFNs in slowing disability accumulation [93].

In terms of tolerability, the more frequent adverse reaction of AZA is leuko/lymphopenia, but, at least in Western countries, it is not associated with a higher incidence of infections and, when not reaching CTC grade 3 toxicity level, it should be indeed considered part of the desired mechanism of action. As for safety, oncogenicity was always considered the main issue of this medication, and a case-control study suggested a dose-response relationship with no significant risk during the first years of treatment and a possible increased risk after about 10 years of continuous therapy [94]. More recently, the risk of malignancy in MS patients after treatment with AZA was reported to be similar to that of the general population, suggesting no or negligible effect of AZA on this adverse event, at least for limited-time exposure [95].

Contraindications include hypersensitivity to active substances, any live vaccine, especially BCG, smallpox, yellow fever, and lactation [96].

### 5.2 Interferons-β (IFN-β)

The first drugs approved by regulatory agencies for MS are Interferons-β (IFN-β), including five preparations used for relapsing-remitting multiple sclerosis (RRMS) and modestly reducing the frequency of relapses [97].

Interferons are naturally occurring cytokines owing a large spectrum of anti-inflammatory activities. Particularly, IFN-β is a class 1 interferon that exerts a complex mechanism of action down-regulating MHC-class II molecule expression on the antigen-presenting cells (APCs). Moreover, its immunomodulating activity depends on reducing the synthesis/secretion of proinflammatory cytokines and increasing the concentration of anti-inflammatory ones. Inhibition of T-cell proliferation and blocking trafficking of inflammatory cells to the central nervous system by down-regulating the integrin very late antigen 4 (VLA-4) [98] play a dramatic role in fighting immunopathogenic events involved in MS [99].

A wide range of recombinant forms of IFN-β are now licensed in RRMS, and two formulations of IFN-β1b (Betaseron^®^/Betaferon^®^, and Extavia^®^) are administered subcutaneously (SC), whereas IFN-β1a is available in two formulations. Avonex^®^ that is injected intramuscularly (IM) and Rebif^®^ which requires a subcutaneous administration.

Despite their good safety and efficacy profile, these drugs have several adverse reactions. The most common are injection site reactions, flu-like symptoms, asthenia, hypersensitivity, myalgia, headache, and liver enzyme elevation [100]. Furthermore, the synthesis and secretion of anti-IFN-β antibodies are more frequent following subcutaneous (>20%) than intramuscular administration (4-7%) [101, 102]. Contraindications involve patients with a history of hypersensitivity to natural or recombinant IFN-β, human albumin or any of the excipients, current severe depression and/or suicidal ideation, and decompensated liver disease.

IFN-β1b was the first drug approved by the FDA for the treatment of MS in 1993 and was granted market authorization in Europe in 1995.

In 2014 FDA and EMA (European Medicines Agency) approved Plegridy^®^ (PEGylated IFNβ-1a) according to the results of an ADVANCE study, a multicentre, randomized, double-blind, parallel-group, placebo-controlled trial (NCT00906399) [103]. The attachment of IFNβ-1a with the polyethylene glycol ensures prolonged and enhanced exposure, maintaining the unaltered efficacy and safety profile [104].

### 5.3 Glatiramer acetate (GA, Copaxone^®^)

Glatiramer acetate (GA, Copaxone^®^) is the acetate salt of a mixture of random polypeptides made of four amino acids (glutamic acid, alanine, tyrosine, and lysine) based on the composition of myelin basic protein (MBP). While the mechanism of action of GA remains a matter of ongoing debate, it seems to act as an altered peptide ligand, cross-reacting with the autoantigen MBP, thus promoting regulatory T-cells (T_reg_) instead of stimulating adverse T-cell autoreactivity [105]. The immunomodulatory action of glatiramer acetate probably originates from indiscriminate binding to MHC-II molecules on APCs, displacing MBP from these binding sites, hence in altered T-cell responses [106]. The mechanism described so far leads to the suppression of myelin-reactive T cells and induces T cells to shift towards the anti-inflammatory T helper (Th)-2 subtype that crosses the BBB and exerts a “bystander suppression” of autoreactive inflammatory T cells in the CNS [107]. Th-2 cells can also have neuroprotective effects by stimulating the production of Brain Derived Neurotrophic Factor (BDNF) [108].

Several pieces of evidence have shown that the administration of GA is associated with decreasing in the amount of B cells, plasmablasts, and memory B cells, as well as a shift from pro- to anti-inflammatory B cell phenotype [109].

Common adverse reactions are injection-site reactions (tenderness, itching, erythema, or induration), as well as mild and transient hypersensitivity reactions of flushing, chest tightness, dyspnoea, palpitations, and anxiety occurring within minutes of the injection in about 10% of patients, lasting a few seconds to several minutes. Regional lymphadenopathy and local lipoatrophy may also occur. GA must not be taken by patients with a history of hypersensitivity either to active agents or to any of the excipients [110].

Copaxone^®^ 20 mg/mL received initial FDA approval in 1996 after the publication of the results of a phase 3 randomized, double-blind, placebo-controlled study of copolymer 1 for relapsing-remitting multiple sclerosis. The trial was provided on 251 patients with RRMS treated for 2 years with GA or placebo subcutaneously injected.

A reduction of about 29% in the annual relapse rate (ARR) was observed in the GA group compared to the placebo group. Significantly more patients on GA improved on the expanding disability status score (EDSS) score, and significantly fewer patients worsened. However, no MRI scans were performed in this trial, except for at one center where patients on GA had significantly fewer gadolinium (Gd) enhancing lesions and reduced brain volume loss compared to patients taking a placebo (NCT00004814) [111-114].

In 2014, the FDA approved Copaxone^®^ 40 mg/mL following the results of a GALA study (a study in subjects with RRMS to assess the efficacy, safety and tolerability of glatiramer acetate injection 40 mg administered three times a week compared to placebo) (NCT01067521) [115].

### 5.4 Teriflunomide (Aubagio^®^)

Teriflunomide (Aubagio^®^) is the active metabolite of leflunomide (a drug licensed for use in patients with rheumatoid arthritis since 1998), acting as immunosuppressor *via* the interference with de-novo synthesis of pyrimidine. It selectively and reversibly inhibits the mitochondrial enzyme dihydroorotate dehydrogenase (DHODH), determining a reduction in the proliferation of T-cells assumed to be autoreactive [116]. Further immunomodulatory implications come from the reduction of nucleotide synthesis, particularly the lack of pyrimidines could cause impaired generation of lipid messengers and malfunction of cell surface molecules [116]. *In vitro* data suggested that another mechanism mediating antiproliferative and anti-inflammatory effects is the inhibitions of protein tyrosine kinase (PTK) activity [117], in particular the Janus-Kinases (JAKs) 1 and 3, involved in intracellular signalling of a number of cytokine receptors [118]. Furthermore, as shown in animal and human studies, teriflunomide has the potential to induce a switch of cytokine profiles from Th1 (proinflammatory) to Th2 (anti-inflammatory) [119].

Considering the above, teriflunomide activity extends beyond the inhibition of DHODH and includes impairing the migratory property of T cells and targets neutrophils and macrophages by modulating their expression of adhesion molecules, migration, adherence, and cytokines secretion [120]. The major advantages of using teriflunomide are that it is administered per os, is well-tolerated and has a good efficacy. Common adverse effects include gastrointestinal manifestations, such as diarrhea, abdominal pain, dyspepsia, nausea and vomiting, increasing liver enzyme levels, susceptibility to infections, hypertension, and hair thinning. Teriflunomide cannot be administered during pregnancy because of its teratogenic consequences, and not even during breastfeeding [121]. Therefore, pregnancy status must be excluded before starting the treatment. Other contraindications include severe hepatic impairment, immunodeficiency states, any bone marrow disorder, dialysis, and drastic hypoproteinaemia [122].

In September 2012 FDA and in 2013 EMA approved teriflunomide (Aubagio^®^) for the treatment of patients with multiple sclerosis, relying on the results of efficacy and safety of two phase 3 trials named TEMSO (NCT00134563) [123, 124] and TOWER (NCT00751881) [125, 126].

### 5.5 Dimethyl Fumarate (DMF, BG-12, Tecfidera^®^)

The immunomodulatory effects of dimethyl fumarate (DMF) are exerted through the activation of the nuclear factor (erythroid-derived 2)-like 2 (Nrf-2) pathway and Nrf2-independent pathways [127]. Nrf-2 represents a transcription factor that maintains redox homeostasis inside the cells; because of its binding to the repressor Keap-1, it is found inactive in the cytoplasm. Migration of Nrf-2 to the nucleus, subsequent to its dissociation from Keap-1, leads to the expression of antioxidants and detoxifying enzymes genes, for instances, NAD(P)H quinone dehydrogenase 1 (NQO-1), glutathione S-transferase-1 (GST-1), and hemoxygenase-1 (HO-1) [128]. Moreover, the Nrf-2 activated pathway induces expansion of FoxP3^+^ T_reg_ and CD56^bright^ natural killer cells, as well as a reduction of CD8^+^ T cells and B cells [129]. Side effects of DMF are nausea, diarrhea, flushing, and abdominal pain. It is contraindicated in case of hypersensitivity to the active substance or to any of the excipients [130].

FDA, in March 2013, and EMA, in January 2014, licensed dimethyl fumarate (Tecfidera^®^) for treating RRMS, after two successful clinical trials: DEFINE (NCT00420212) [131, 132], a randomized, multicentre, double-blind, placebo-controlled, dose-comparison study performed in order to determine the efficacy and safety; and CONFIRM a randomized, multicentre, placebo-controlled and active reference (glatiramer acetate) comparison study having the same aim (NCT00451451) [133, 134]. Both studies have demonstrated that DMF can significantly improve MS clinical parameters by reducing the relapse rate and the EDSS in comparison with placebo.

### 5.6 Fingolimod (FTY720, Gilenya^®^)

Fingolimod (FTY720), derived from myriocin, a metabolite of the fungus Isaria sinclairii [135], was the first line oral therapy for RRMS, which acts as a sphingosine-1-phosphate (S1P) receptor antagonist preventing the egression of lymphocytes from secondary lymphatic tissues inhibiting the entry of autoreactive lymphocytes into the central nervous system. Furthermore, it non-selectively depredates the S1P1 receptors on T cells besides its internalization, reducing their responsiveness to chemotactic signals [136]. Most common adverse reactions include headache, elevation of liver enzymes, diarrhea, cough, influenza, sinusitis, back pain, bradycardia, and less frequently, first or second-degree atrioventricular block for which patients should be monitored for at least 6 hours after the first dose [137]. FTY720 cannot be administered in patients suffering from immunodeficiency states or being at heightened risk for opportunistic infections, nor in case of acute infestations. Contraindications also encompass hepatic failure and active malignancies. Important warnings regard those patients who have been affected by myocardial infarction, unstable angina pectoris, stroke/transient ischemic attack, decompensated heart failure requiring hospitalisation, or New York Heart Association (NYHA) class III or IV heart failure in the last 6 months, in addition to patients who have Mobitz type II second or third-degree atrioventricular (AV) block, or sick sinus syndrome, unless the patient has a pacemaker implanted. The presence of severe cardiac arrhythmias requiring treatment with class Ia or class III anti-arrhythmic medicinal products, as well as a baseline QTc interval ≥ 500 msec, contraindicated the drug administration [137].

Two licensing double-blind, randomized, phase 3 trials (FREEDOMS (NCT00289978) [138] and TRANSFORMS (NCT00340834) [139]) [140] supported a previous phase 2 trial (NCT00333138) [141, 142] that led to the drug's approval by FDA in 2010 and by EMA in March 2011.

### 5.7 Siponimod (BAF312, Mayzent^®^)

Siponimod (BAF312) is an oral selective sphingosine 1-phosphate (S1P) receptor modulator (S1P1 and SIP5). Compared with fingolimod, siponimod has a novel chemotype and does not need to be phosphorylated *in vivo*. It has a mean half-life of approximately 30 hours and typically washes out within 6.3 days after discontinuation. Modulation of S1P1 on peripheral lymphocytes inhibits their egress from lymph nodes and, therefore, infiltration of the CNS [143, 144]. Moreover, siponimod crosses the BBB [145], and preclinical data have shown a reduction of central nervous system inflammation and, in addition, indicate effects on repair mechanisms *via* modulation of S1P_1_ on astrocytes and S1P_5_ on oligodendrocytes [146, 147].

The most common adverse reactions include headache (15%), hypertension (12.6%), dizziness, lowered heart rate, and increased risk of upper respiratory infections. In addition to the contraindications listed for fingolimod, this medication cannot be taken by patients with a history of progressive multifocal leukoencephalopathy or cryptococcal meningitis [148].

Siponimod approval process began in 2016 with the publication of the results of the phase 2 study bold (A Dose Blinded Extension Study to the CBAF312A2201 Study to Evaluate Long-term Safety, Tolerability and Efficacy of BAF312 Given Orally Once Daily in Patients With Relapsing-remitting Multiple Sclerosis) (NCT01185821) [149], which enrolled 184 participants with RRMS. The study consisted of a two-year dose-blinded phase during which patients received one of five doses of siponimod (10, 2, 1.25, 0.5 or 0.25 mg), following which patients were switched to open-label treatment with siponimod 2 mg for approximately a further three years, with the aim to provide data on long term safety, tolerability, and efficacy. It was observed that siponimod reduced the number of brain lesions assessed on MRI by more than 80% and reduced the frequency of relapse when compared to placebo. The results of the study, which was an extension of the bold study, showed that disease activity assessed by MRI activity and relapse frequency remained low, particularly in the 1.25, 2, and 10 mg treatment groups. The authors concluded that siponimod treatment results in a reduction in disease activity. Therefore, a phase 3 study was encouraged [147].

After that, siponimod’s efficacy and safety have been further evaluated with a phase 3 trial, named EXPAND (Exploring the Efficacy and Safety of Siponimod in Patients with Secondary Progressive Multiple Sclerosis) (NCT01665144) [150], which enrolled 1651 subjects with SPMS. Study participants received oral siponimod or placebo once daily for up to three years. The EDSS was evaluated every 3 months, and the researchers found that 32% of those taking placebo had an increase in disability confirmed for 3 months during the study, compared to 26% of those taking siponimod. Therefore, there was a 21% reduction in the risk of progression for siponimod group. Further analysis highlighted a 33% reduction in the risk of progression for those with “active” SPMS (defined as those who had relapsed in the two years prior to the start of the trial). Furthermore, siponimod proved to be more effective than placebo in other assessments used in the study, such as reduction in brain atrophy and reduction in lesions volume on MRI. The main side effects observed to a greater extent in the siponimod treatment group during this study were: a decrease in white blood cells, an increase in liver enzymes, a decrease in heart rate at the start of treatment, macular oedema, an increase in blood pressure and convulsions [151].

The results of these two trials led to the drug’s approval by the FDA in March 2019 and by EMA in January 2020.

### 5.8 Ozanimod (Zeposia^®^)

Ozanimod is a quite novel oral sphingosine-1-phosphate receptor (S1PR) modulator that selectively targets S1P1 and S1P5 with high affinity, thus preventing circulating autoreactive lymphocytes from entering the CNS from peripheral tissues, as well as reducing their presence in the bloodstream [152].

Ozanimod, acting as a functional antagonist of the aforementioned receptors, determines a sustained internalisation and degradation of S1P1 receptors on lymphocytes, which inhibit their egression from lymph nodes and, as a consequence, their trafficking to inflamed tissue sites [153]. A rapid dose-dependent reduction in absolute lymphocyte count (ALC) occurs in patients taking ozanimod, with the greatest decrease in lymphocyte subsets expressing cytokine receptor 7 (CCR7+), as well as central memory cells being more affected than effector memory cells [154].

Before initiating treatment with ozanimod, patients should undergo certain assessments, which include a complete blood count, electrocardiogram, and liver function tests. In those who suffer from uveitis or macular oedema, an ophthalmic examination should be performed too. Given the potential interactions with other treatments, a drug history, including current and prior medications, should be accurately collected. As ozanimod may increase the risk of infections because of lymphocyte depletion, a test for antibodies to varicella zoster virus is also required, and any live-attenuated vaccines should be avoided up to 1 month prior to initiating therapy with ozanimod. Moreover, ozanimod is contraindicated in patients who have been affected by myocardial infarction, unstable angina, stroke, transient ischemic attack, decompensated heart failure requiring hospitalisation, or NYHA class III or IV heart failure in the last 6 months, in addition to patients who have Mobitz type II second or third-degree atrioventricular (AV) block, sick sinus syndrome, or sino-atrial block, unless the patient has a pacemaker implanted. Should not be treated with ozanimod for patients with severe untreated sleep apnoea or concomitantly under therapy with monoamine oxidase inhibitors (iMAO) [155]. Transitory AV conduction delays and reduced heart rate may occur at the beginning of therapy. Therefore, the suggested titration scheme should be respected. Patients with hypersensitivity, active chronic infestations, active malignancies, and severe hepatic impairment must avoid taking the drug. Women of childbearing potential not using effective contraception cannot be administered with ozanimod. The most commonly reported adverse reactions (> 5%) are nasopharyngitis, alanine aminotransferase (ALT), and gamma-glutamyl transferase increase [156].

Ozanimod’s approval was based on positive results from RADIANCE (A Phase 2/3, Multi-centre, Randomized, Double-blind, Placebo-controlled (Part A) and Double-blind, Double-dummy, Active-controlled (Part B), Parallel Group Study to Evaluate the Efficacy and Safety of RPC1063 Administered Orally to Relapsing Multiple Sclerosis Patients) (NCT02047734) [157] and SUNBEAM (A Phase 3, Multi-Centre, Randomized, Double-Blind, Double-Dummy, Active Controlled, Parallel Group Study To Evaluate The Efficacy And Safety Of RPC1063 Administered Orally To Relapsing Multiple Sclerosis Patients) (NCT02294058) trials [158]. In the phase 2 portion (Part A) of RADIANCE, reported separately as NCT01628393, 258 patients with RRMS diagnosed per the 2010 McDonald criteria, were randomized to ozanimod 0.5 mg, ozanimod 1 mg, or placebo. During the 24 weeks, those treated with both ozanimod 0.5 or 1 mg have shown significant reductions in mean cumulative number of gadolinium-enhancing lesions and new or enlarging T2-hyperintense lesions compared to placebo [159, 160].

At week 24, patients could enrol in a dose-blinded extension study, in which participants previously randomized to ozanimod, continued therapy with the same dose, and participants originally administered a placebo were switched to either ozanimod 0.5 or 1 mg [152].

The 2-year extension study was concluded by 89.6% (223) participants. Patients who received continuous ozanimod maintained the same efficacy, and those initially assigned to placebo showed a similar reduction in the mean number of gadolinium-enhancing lesions.

The RADIANCE phase 3 trial consisted of a 24-month double-blind, double-dummy, active-controlled, parallel-group clinical study with the purpose of assessing the safety and efficacy of once-daily ozanimod 0.5 and 1 mg *versus* weekly intramuscular interferon β-1a 30 μg. Adjusted annualized relapse rates were established as the primary endpoint and were significantly lower in patients treated both with ozanimod 0.5 and 1 mg (0.17 and 0.22, respectively) compared with those treated with intramuscular interferon β-1a (0.28). Moreover, loss of whole-brain volume, cortical grey matter, and thalamic volume was found to be decreased in both ozanimod doses [159].

The SUNBEAM phase III trial was conducted concurrently with RADIANCE phase 3, also comparing daily oral ozanimod 0.5 mg or 1 mg with weekly intramuscular interferon β-1a 30 μg over at least 12 months of treatment. The adjusted ARR was 0.18 for the ozanimod 1 mg group, 0.24 for the ozanimod 0.5 mg group, and 0.35 for the interferon β-1a group [161].

Both SUNBEAM and RADIANCE phase 3 clinical trials concluded that either low or high-dose ozanimod was as effective as interferon β-1a in reducing active disease in relapsing MS.

The results collected from RADIANCE phase 3 and SUNBEAM led to the approval of ozanimod on 25 March 2020 by the FDA for the treatment of relapsing forms of multiple sclerosis, including clinically isolated syndrome, relapsing-remitting disease, and active secondary progressive disease, in adults, as well as the authorization on 20 May 2020 by EMA for RRMS.

### 5.9 Ponesimod (Ponvory^®^)

Ponesimod is a selective, orally active, rapidly reversible S1P1 receptor modulator. Ponesimod exerts its immunomodulating activity *via* the functional antagonism of the S1P1 receptor expressed on lymphocytes, thus preventing their egression from lymph nodes and, as a result, their circulation in the blood flow and migration to the sites of inflammation [162]. Lymphocyte subpopulations exhibit a different sensibility to ponesimod, being more affected CD3+ expressing cells, as well as CD20+ B cells, with a rapidly transient decrease count in the peripheral bloodstream, whilst no modification has been found on natural killer cells (CD16+) [163].

The most commonly reported adverse drug reactions are nasopharyngitis (19.7%), alanine aminotransferase increase (17.9%) and upper respiratory tract infection (11%) [164]. The same contraindications and pre-treatment evaluations as observed for ozanimod apply to the ponesimod [165].

The phase II double-blind, placebo-controlled, dose-finding study performed on 464 adult patients with RRMS in order to evaluate efficacy, safety, and tolerability of three once-daily different doses of ponesimod, showed a dose-dependent reduction in the mean cumulative number of new T1 gadolinium-enhanced lesions, set as a primary endpoint. More specifically, the reduction was by 43% in the 10 mg group, by 83% with 20 mg and by 77% with 40 mg ponesimod, compared to placebo (NCT01006265) [166, 167].

The OPTIMUM phase 3 trial (Multicentre, Randomized, Double-blind, Parallel-group, Active-controlled, Superiority Study to Compare the Efficacy and Safety of Ponesimod to Teriflunomide in Subjects With Relapsing Multiple Sclerosis) enrolled 1133 participants diagnosed with RRMS, aiming at comparing the efficacy and safety of once-daily 20 mg ponesimod to teriflunomide 14 mg over 108 weeks. Regarding the primary endpoint which was the ARR, ponesimod was superior to teriflunomide in reducing the incidence of relapses by 30.5% [168].

Based on the results of the OPTIMUM phase 3 study (NCT02425644) [169], ponesimod received approval on 18 March 2021 by the FDA and on 19 May 2021 by EMA for the treatment of relapsing forms of MS.

### 5.10 Natalizumab (Tysabri^®^)

Natalizumab is a humanized recombinant IgG4 monoclonal antibody (mAb) targeting the α4-integrin molecule, a component of very late antigen-4 (VLA-4), expressed on lymphocytes preventing binding to the ligand vascular cell adhesion molecule (VCAM) found on endothelial cells surfaces. This blocks the adhesion and subsequent extravasation of lymphocytes across the BBB, reducing CNS inflammation [97, 170]. Integrins are cell-surface glycoproteins that facilitate cell-matrix adhesion and mediate leukocyte rolling and adhesion to the endothelium prior to extravasation [171].

Two phase 3 clinical trials led to natalizumab approval by regulatory agencies: AFFIRM and SENTINEL studies. AFFIRM (Safety and Efficacy of Natalizumab in Relapsing-Remitting Multiple Sclerosis) study enrolled 942 RRMS patients to receive either natalizumab (300 mg) or placebo intravenously (IV) every 4 weeks for up to 116 weeks (NCT00027300) [172]. The clinical relapse rate was reduced by 68%, and the risk of sustained progression of disability was reduced by 42% over 2 years. MRI activity was reduced by 92% in the natalizumab-treated group [173, 174]. SENTINEL study recruited 1,171 patients who had at least one relapse whilst on IFN-β1a therapy in the previous 12 months. They received intramuscular IFN-β1a in combination with 300 mg of natalizumab or placebo (NCT00030966) [175]. The outcome measures were superimposable to those of the AFFIRM study and showed that combination therapy with natalizumab yielded a 55% reduction in the ARR and a 24% reduction in the risk of sustained disability progression at 2 years [176]. The publication of the safety and efficacy results of these two studies led to drug approval by the FDA in 2004 and by EMA in 2006.

More recently, a subcutaneous formulation of Tysabri^®^ has been developed, although limited data for its administration in treatment-naïve patient populations are available [177].

Common adverse drug effects include injection-site reactions during infusion, increased risk of developing infections (especially in the urinary and upper respiratory tract), alteration of haematochemical parameters (such as number of white blood cells, red blood cells, platelets, or liver function enzymes), headache, fatigue, joint pain, vomiting, and hives.

Contraindications include hypersensitivity to the active substance or to any of the excipients, PML, increased risk for opportunistic infections, and active malignancies. Natalizumab cannot be taken in combination with other DMTs [177].

Shortly after natalizumab approval, the drug was withdrawn from the market after three patients developed Progressive Multifocal Leukoencephalopathy (PML), a life-threatening CNS-demyelinating disease caused by infection of oligodendrocytes with the John Cunningham virus (JCV) [178]. In immunocompetent subjects, JCV remains latent, not causing the disease, whereas PML more frequently affects immunosuppressed individuals, such as patients with acquired immunodeficiency syndrome (AIDS). In patients receiving natalizumab the disease is apparently related to the forced migration of cells harbouring JCV out of the bone marrow and the upregulation of gene products in B cell maturation that also promotes virus growth [179].

In 2006, natalizumab was reintroduced to the market with a black-box warning about PML risks.

### 5.11 Alemtuzumab (Lemtrada^®^)

Alemtuzumab is a humanized monoclonal IgG1-antibody that targets CD52, a surface glycoprotein with partially unknown functions predominantly expressed (> 95%) on T (CD3^+^) and B (CD19^+^) cells [180, 181]. Lower expression levels are found on natural killer (NK) cells, monocytes, macrophages, and eosinophils, whereas plasma cells, neutrophils, and haematological stem cells show little or no expression [182].

As written above, the role of CD52 is still vaguely understood [183]. However, it seems to be involved in T lymphocyte migration and co-stimulation [184, 185].

The bind between alemtuzumab and CD52 leads to a rapid and long-lasting depletion of CD52-positive cells by antibody-dependent cell-mediated cytotoxicity (ADCC) and complement-dependent cytolysis (CDC), followed by a slow repopulation arising from unaffected hematopoietic-precursor cells [186]. Both quantitative and qualitative changes in the immune-cell repertoire are observed, which might contribute to a rebalancing of autoimmune processes through changes in the number, percentages, and properties of some lymphocyte subgroups after treatment, the increased presence of regulatory T cell subgroups, the increased presence of T and B cell memory, and transient effects on the components of innate immunity (*e.g*., neutrophils, macrophages, NK cells) [187]. Alemtuzumab depletes circulating T and B lymphocytes after each treatment cycle, reaching the lowest values 1 month after a course of treatment. Lymphocytes repopulate over time with a recovery of B cells that is usually completed within 6 months. CD3^+^ and CD4^+^ cell counts reach normal values more slowly but generally do not return to baseline within 12 months after treatment. About 40% of patients had a total lymphocyte count that reached the lower limit of the normal range (LLN) within 6 months after each treatment course, and approximately 80% of patients had a total lymphocyte count reaching the LLN within 12 months after each cycle [187].

Adverse drug reactions include autoimmune-associated diseases, infusion-associated reactions (IARs), infections (especially upper respiratory tract infections and urinary tract infections), heart disease, and lymphoproliferative disorders associated with Epstein-Barr virus. Malignancies such as thyroid cancer, melanoma, and melanoma-*in-situ*, as well as lymphoproliferative disorders, have been reported. A history of arterial dissection of the cervicocephalic arteries, stroke, angina pectoris, or myocardial infarction contraindicates drug intake. Other limitations involve hypersensitivity, uncontrolled hypertension, severe active infections including HIV, coagulopathy, on anti-platelet or anti-coagulant therapy, and other concomitant autoimmune disorders besides MS [187].

The Alemtuzumab approval process began in 2008 with the publication in The New England Journal of Medicine (NEJM), of the results of the phase 2 study CAMMS223 [188], which enrolled 334 patients with RRMS. This study compared two different doses of alemtuzumab with interferon β1-a administered three times a week. Data analysis showed that after 36 months, people treated with alemtuzumab 12 mg/day had a reduced risk of developing relapses of about 69% and a reduction of about 76% of the accumulation of sustained disability compared to the control group treated with interferon. Concluding that in patients with early, relapsing-remitting multiple sclerosis, alemtuzumab was more effective than interferon β-1a, but it was associated with autoimmunity, most seriously manifesting as immune thrombocytopenic purpura [189]. In 2012, Coles *et al.* published the results of the 5-year extension of the CAMMS223 study, concluding that alemtuzumab remained significantly more efficacious than IFNβ-1a, with a safety profile consistent with previous reports [190].

Alemtuzumab’s efficacy and safety have been further evaluated with two phase 3 trials, named CARE-MS I (Comparison of Alemtuzumab and Rebif^®^ Efficacy in Multiple Sclerosis, Study One) [191] (NCT00530348) and CARE-MS II (Comparison of Alemtuzumab and Rebif^®^ Efficacy in Multiple Sclerosis, Study Two) [192] (NCT00548405).

The purpose of the CARE-MS I study was to assess efficacy and safety of first-line alemtuzumab, administered in two annual courses, once at the beginning of the study (12 mg per day IV infusion on 5 consecutive days) and again 1 year later (12 mg per day IV infusion on 3 consecutive days), compared with interferon β-1a (44 microgram (mcg) subcutaneously administrated three times a week for 24 months) in 581 treatment-naïve patients with RRMS. The two-year study results showed that alemtuzumab reduced the frequency of relapses by about 55% compared to patients taking interferon. In addition, 78% of people taking alemtuzumab did not relapse during the two-year study period compared to 59% of the interferon treatment group. There was no significant effect on disability progression, indeed 8% of patients taking alemtuzumab and 11% of those taking interferon showed a worsening in disability [193].

The CARE-MS II study enrolled 667 participants who had received an adequate trial of disease-modifying therapies but experienced at least one relapse during prior treatments and who met a minimum severity of disease as measured by MRI. The study aim was to establish the efficacy and safety of two different doses of alemtuzumab (12 or 24 mg per day intravenously administrated for 5 consecutive days at month 0, followed by alemtuzumab 12 or 24 mg per day intravenously administrated for 3 consecutive days at month 12) as a treatment for RRMS, in comparison with subcutaneous interferon β-1a (44 mcg subcutaneously (SC) administrated three times a week for 24 months). The study results showed that the relapse frequency of patients taking alemtuzumab was reduced by 49% compared to those taking interferon, and a significant reduction in the accumulation of sustained disability assessed at 6 months was also observed (42%). Sixty-five percent of participants treated with alemtuzumab, had not relapsed during the two-year treatment period compared to the interferon group (47%). In addition, there was a small improvement in EDSS score in the alemtuzumab treatment group compared to a small worsening in EDSS score in the interferon group [194].

In both CARE-MS I and II studies, alemtuzumab significantly reduced the frequency of relapses over two years to SC IFN-β-1a; remarkably improved MRI outcomes, including gd-enhancing lesions and new or enlarging T2 lesions in the alemtuzumab cohort compared to the IFN-β-1a cohort and reduced the rate of brain-volume loss [107]. The publication of the safety and efficacy results of these two studies led to drug approval by EMA in 2013 and by FDA in 2014.

### 5.12 Rituximab (RTX, MabThera^®^)

Rituximab is a genetically engineered chimeric mouse/human monoclonal antibody representing a glycosylated immunoglobulin with human IgG1 constant regions and murine light-chain and heavy-chain variable region sequences. The antibody is produced by mammalian (Chinese hamster ovary) cell suspension culture and purified by affinity chromatography and ion exchange, including specific viral inactivation and removal procedures.

Rituximab binds specifically to the transmembrane antigen, CD20, a non-glycosylated phosphoprotein, located on pre-B and mature B lymphocytes. CD20 is found on both normal and malignant B cells, but not on haematopoietic stem cells, pro-B cells, normal plasma cells, or other normal tissue. This antigen does not internalise upon antibody binding and is not shed from the cell surface. CD20 does not circulate in the plasma as a free antigen and, thus, does not compete for antibody binding. The Fab domain of rituximab binds to the CD20 antigen on B lymphocytes and the Fc domain can recruit immune effector functions to mediate B cell lysis. Possible mechanisms of effector-mediated cell lysis include CDC resulting from C1q binding, and ADCC mediated by one or more of the Fcγ receptors on the surface of granulocytes, macrophages, and NK cells. Rituximab binding to CD20 antigen on B lymphocytes has also been demonstrated to induce cell death *via* apoptosis [195].

Common adverse drug reactions include infections and infestations (such as bacterial infections and viral infections), blood lymphatic system alterations (neutropenia, leucopoenia, anaemia, and thrombocytopenia), immune system disorders (infusion-related reactions), metabolism and nutrition impairments, psychiatric, nervous system, eye, cardiac, and vascular diseases [195].

Drug safety and efficacy in MS was first evaluated in 2004 in phase II, randomized, double-blind, parallel-group, placebo-controlled, multicentre study entitled “A Phase II, Randomized, Double-Blind, Parallel-Group, Placebo-Controlled, Multicentre Study to Evaluate the Safety and Efficacy of Rituximab (Mabthera/Rituxan) in Adults With Relapsing-Remitting Multiple Sclerosis” (NCT00097^188^]. The study, lasting 48 weeks, enrolled 104 patients with RRMS, 69 received 1000 mg of intravenous rituximab, and 35 received placebo on days 1 and 15. The primary endpoint was the total count of gadolinium-enhancing lesions detected on MRI scans of the brain at weeks 12, 16, 20, and 24. Clinical outcomes included safety, the proportion of patients who had relapsed, and the annualized rate of relapse. The study results showed that patients who received rituximab had reduced counts of total gadolinium-enhancing lesions at weeks 12, 16, 20, and 24 (*P <* 0.001) and of total new gadolinium-enhancing lesions over the same period (*P <* 0.001); these results were sustained for 48 weeks (*P <* 0.001). As compared with patients in the placebo group, the proportion of patients in the rituximab group with relapses was significantly reduced at week 24 (14.5% *vs*. 34.3%, *P =* 0.02) and week 48 (20.3% *vs*. 40.0%, *P =* 0.04). More patients in the rituximab group than in the placebo group had adverse events within 24 hours after the first infusion, most of which were mild-to-moderate events; after the second infusion, the numbers of events were similar in the two groups. Given the results of the study, Hauser *et al.* concluded that a single course of rituximab reduced inflammatory brain lesions and clinical relapses for 48 weeks. This trial was not designed to assess long-term safety or to detect uncommon adverse events. The data provide evidence of B-cell involvement in the pathophysiology of RRMS [196, 197].

Furthermore, always in 2004, rituximab was also evaluated in a phase II/III randomized, double-blind, parallel group, placebo controlled, multicentre study to evaluate the safety and efficacy of rituximab in adults with PPMS (NCT00087529). This study, using 2:1 randomization, enrolled 439 participants who received two doses of 1000 mg intravenous rituximab or placebo infusions every 24 weeks, through 96 weeks (4 courses). The primary endpoint was time to confirmed disease progression (CDP), a prespecified increase in EDSS sustained for 12 weeks. Secondary endpoints aim to assess the change from baseline to week 96 in T2 lesion volume and total brain volume on MRI scans. Trial results highlighted that differences in time to CDP between rituximab and placebo did not reach significance (96-week rates: 38.5% placebo, 30.2% rituximab; *P =* 0.14). From baseline to week 96, rituximab patients had less (*P <* 0.001) increase in T2 lesion volume; brain volume change was similar (*P =* 0.62) to placebo. Subgroup analysis showed time to CDP was delayed in rituximab-treated patients aged < 51 years (HR = 0.52; *P =* 0.010), those with gadolinium-enhancing lesions (HR = 0.41; *P =* 0.007), and those aged < 51 years with gadolinium-enhancing lesions (HR = 0.33; *P =* 0.009) compared with placebo. Adverse events were comparable between groups; 16.1% of rituximab and 13.6% of placebo patients reported serious events. Serious infections occurred in 4.5% of rituximab and < 1.0% of placebo patients. Infusion-related events, predominantly mild to moderate, were more common with rituximab during the first course and decreased to rates comparable to placebo on successive courses. Hawker *et al.* concluded that, although time to CDP between groups was not significant, overall subgroup analyses suggest selective B-cell depletion may affect disease progression in younger patients, particularly those with inflammatory lesions [198, 199].

Despite clinical trials conducted in patients with MS to evaluate the safety and efficacy of the molecule, rituximab has not been approved for this indication by regulatory agencies. Therefore, it still remains an off-label treatment.

### 5.13 Ocrelizumab (Ocrevus^®^)

Ocrelizumab is a recombinant humanised IgG1 anti-CD20 antibody that binds avidly to CD20, a transmembrane phosphoprotein expressed on the surface of mature B cells [200]. CD20 is the same target as rituximab. However, ocrelizumab is directed against a different but overlapping epitope of the extracellular domain of CD20 and leads to a dose-dependent depletion of B cells *via* the ADCC mechanism. Moreover, rituximab is a chimeric antibody and acts predominantly *via* CDC [201, 202]. Due to the human origin of this monoclonal antibody, it is expected to be less immunogenic and therefore less likely to cause infusion reactions or induce the neutralising antibody formation [203].

Ocrelizumab selectively depletes CD20-expressing B cells, preserving pre-existing humoral immunity and the capacity for B cell reconstitution. B cell depletion is associated with a potent interruption in B cell trafficking from the periphery to the CNS, reduced B cell antigen presentation to T cells, modulation of proinflammatory cytokine secretion by B cells, and reduced activation and differentiation to immunoglobulin secreting plasmablasts [204, 205].

Two randomized, double-blind, double-dummy, parallel-group phase 3 trials led to the drug’s approval by FDA in March 2017 and by EMA in 2018, OPERA I (A Study of Ocrelizumab in Comparison With Interferon Beta-1a (Rebif^®^) in Participants With Relapsing Multiple Sclerosis) (NCT01247324) [206] and OPERA II (A Study of Ocrelizumab in Comparison With Interferon Beta-1a (Rebif^®^) in Participants with Relapsing Multiple Sclerosis) (NCT01412333) [207]. The purpose of these two studies was to define the efficacy and safety profile of ocrelizumab in comparison with interferon β -1a in 1.656 participants with RRMS. Patients have been randomised to receive either ocrelizumab 600 mg or matching placebo IV as 300 mg infusions on days 1 and 15 for the first dose and as a single infusion of 600 mg for all subsequent infusions every 24 weeks, with placebo injections matching interferon β -1a SC three times per week or interferon β-1a 44 mcg SC injections three times per week (with placebo infusions matching ocrelizumab infusions every 24 weeks). The results of the two studies, published by Hauser *et al.* in the NEJM, showed that ocrelizumab significantly reduced the annualised relapse rate (the primary objective of both studies) by about 50% compared to interferon β-1a over a two-year period. In addition, secondary objectives, such as significantly reducing the risk of disability progression, were also achieved. Finally, ocrelizumab also significantly reduced signs of inflammation and the appearance of new lesions capturing contrast medium on MRI [208].

Montalban *et al.* published the result of the ORATORIO study, a randomized, parallel-group, double-blind, placebo-controlled trial, which aimed to evaluate the efficacy and safety of ocrelizumab *versus* placebo in 732 participants with PPMS (NCT01194570) [209].

Ocrelizumab significantly reduced the risk of disability progression (by about 24%). This result was also maintained at 24 weeks (secondary endpoint) with a 25% risk reduction compared to the placebo group. Finally, it decreased the volume of hyperintense T2 lesions and reduced the rate of brain atrophy by 17.5% compared to placebo [210].

The most important and frequently reported adverse reactions were infusion-related reactions and infections.

Contraindications involve hypersensitivity to the active substance or to any of the excipients, current active infection, patients in a severely immunocompromised state, and known active malignancies [211].

On 16 June 2020, the European Medicines Agency, following a positive opinion from the Committee for Medicinal Products for Human Use (CHMP), approved a new infusion time for ocrelizumab following the publication of the results of the ENSEMBLE PLUS, a randomized double-blind trial (NCT03085810) [212], which demonstrates that the two-hour ocrelizumab infusion time and the conventional 3.5-hour time resulted in infusion-related reactions of comparable frequency and severity both in patients who were treated with a shorter infusion time and patients treated with conventional infusion time [213].

### 5.14 Ofatumumab (Kesimpta^®^)

Ofatumumab is a recombinant fully human anti-CD20 monoclonal immunoglobulin G1 antibody, which binds to a region of the CD20 different from that of other anti-CD20 antibodies [214]. While the precise mechanism of action of ofatumumab is not well defined, it is known that the binding between the FAB portion of ofatumumab and CD20 leads to B cell (and T cell) depletion *via* complement-mediated CD20^+^ B cell lysis (CDC) and ADCC [215, 216].

Ofatumumab is the first B cell-targeting therapy that is intended for subcutaneous self-injection at home (following initial training by a healthcare professional). The approval process of SC ofatumumab in patients with RRMS began in 2016, relying on two identical, multicentre, randomized, double-blind, double-dummy, active-comparator controlled phase 3 trials, ASCLEPIOS I and II (Efficacy and Safety of Ofatumumab Compared to Teriflunomide in Patients With Relapsing Multiple Sclerosis I and II). The aim of these two trials was to compare the efficacy and safety of ofatumumab administered subcutaneously every 4 weeks *versus* teriflunomide administered orally once daily for 2.5 years, in a total of 1,885 patients with RRMS. The dose regimen for ofatumumab for this study was a loading dose of 20 mg at days 1, 7, and 14 followed by a maintenance dose of 20 mg administered every 4 weeks starting at week 4 (NCT02792218; NCT02792231) [217, 218]. These studies showed that the mAb reduced relapses by 50-59% and disability progression by about 30% compared to teriflunomide. Ofatumumab also reduced the number of new lesions on MRI compared to teriflunomide (by about 96% in the number of T1-weighted gadolinium-captured lesions and by 83% in the rate of new or enlarged T2-weighted lesions).

Adverse events were reported in 83.6% of ofatumumab recipients and 84.2% of teriflunomide recipients. The most commonly reported AEs were systemic injection-related reactions, nasopharyngitis, headache, injection-site reactions, upper respiratory tract infections, and urinary tract infections [219, 220]. Known active malignancies, immunocompromised states, severe infections until resolution, and hypersensitivity contraindicate drug use [216].

The publication of the safety and efficacy results of these two studies led to drug’s authorisation by FDA, in August 2020, for the treatment of relapsing-remitting multiple sclerosis and secondarily progressive active multiple sclerosis in adult patients and by EMA, in April 2021, for the treatment of relapsing forms of multiple sclerosis with clinically or neuroimaging evidence of disease activity.

### 5.15 CNS Bioavailable Immunosuppressive DMTs

#### 5.15.1 High-dose Immunosuppression Followed by Autologous Hematopoietic Stem Cell Transplantation (AHSCT)

AHSCT is a haematological procedure that has been increasingly used over the last two decades as a therapeutic strategy for severe treatment-resistant autoimmune diseases [221]. In this setting, MS represents the most frequent indication, with more than 1,500 patients treated and reported to the European Society for Bone and Marrow Transplantation (EBMT) Registry [222].

AHSCT induces the ablation of the immune system through the administration of high-dose chemotherapy. This is followed by the reconstitution of the immune system, promoted by the reinfusion of hematopoietic stem cells (HSCs) previously collected from the individual himself. AHSCT encompasses the following four steps: (i) HSCs mobilization and (ii) collection, (iii) administration of conditioning chemotherapy, (iv) HSCs reinfusion. HSCs are usually mobilized from the bone marrow to the peripheral blood with the association of chemotherapy (*e.g*., cyclophosphamide (CY), 2-4 g/m^2^ body surface area) and hematopoietic growth factors, usually granulocyte-colony stimulating factor (G-CSF). Ten to fifteen days after the mobilization, HSCs are collected with leukapheresis (optimal target of 5x10^6^ CD34^+^ cells/Kg) and cryopreserved until the transplant, performed as an inpatient procedure on average one month later. In this stage, patients receive high-dose chemotherapy (conditioning regimen) that eradicates the immune system and induces bone marrow aplasia. Different combinations of chemotherapy drugs and serotherapy (anti-thymocyte globulin - ATG, or monoclonal antibodies such as rituximab and alemtuzumab) are used as conditioning regimens; these are classified by the EBMT guidelines according to the level of immunosuppression induced into low-, intermediate- and high-intensity regimens [222]. The use of intermediate-intensity regimens is recommended for the treatment of MS [222], and the two most used protocols are the followings: CY 200 mg/Kg + ATG, defined as “lymphoablative”, as the reinfusion of HSCs is not necessary to promote recovery from bone marrow aplasia, even if it speeds up the immune repopulation; and BEAM (carmustine, etoposide, cytarabine and melphalan) + ATG, defined as “myeloablative”, *i.e*., the reinfusion of HSCs is mandatory as a rescue from bone marrow aplasia. After the administration of chemotherapy, HSCs are thawed and reinfused (day 0 of the transplant), and this is followed by haematological recovery with engraftment, usually within two weeks. In this stage, patients receive supportive treatments, including red blood cells and platelet transfusions and antimicrobial therapies. Patients are then discharged home on average within one month from day 0.

The timing of immune cell recovery differs across cell populations: NK cells, B cells and CD8+ T cells repopulate in the first weeks to six months, whereas CD4+ T cells require up to two years [223]. Immune cell reconstitution is prompted by two different mechanisms: (i) early homeostatic expansion of residual T cells over the first six months, followed by (ii) *the novo* generation of T cell clones prompted by thymopoiesis, this latter most effective 1-2 years after AHSCT. This process is characterized by deep changes in the immune system, including renewal of the T cell receptor repertoire with the disappearance of clonal restrictions observed before transplant, expansion of peripheral immunoregulatory cells, modifications in patterns of cytokines production towards a less inflammatory environment, and normalization of the expression of genes involved in the regulation of T cell activity, immune regulatory networks and apoptosis [224]. In addition to these mechanisms, most of the drugs administered during conditioning (such as busulfan, CY, carmustine, and etoposide) cross the BBB [225], thus potentially affecting CNS compartmentalized inflammation. Partially supporting this hypothesis, the observation that CSF oligoclonal bands disappear over long-term follow-up after AHSCT [226] and neurofilament-light chains normalize in the CSF [227].

The safety profile of AHSCT has greatly improved over time, and a remarkable reduction in transplant-related mortality to an estimated 0.3% was observed over the last two decades [223]. Common early toxicities are expected in most cases, such as transient alopecia, mucositis, opportunistic infections, and viral reactivations (namely EBV and CMV). Long-term complications include secondary autoimmune diseases, which are reported in 2-18% of cases, with differences across regimens (higher risk for the use of *ex vivo* CD34^+^ cells selection or alemtuzumab) [228, 229]; infertility and secondary amenorrhea, with higher risk for females aged >35 years-old [230, 231]; and the risk for secondary neoplasms, although the causal relationship in this latter case is not conclusive due to the small number of incident cancers observed and the potential contribution of previous treatments [232].

The effectiveness of AHSCT in MS was demonstrated by several prospective and retrospective studies, overall showing suppression of new inflammatory activity (relapses and new/gadolinium enhancing lesions at MRI) in 80-100% of cases at long-term follow-up [227, 233-237]. On the other hand, heterogeneous results were observed on disability accrual across studies, mostly depending on the relative proportion of relapsing *vs.* progressive patients included and on baseline EDSS, being the benefit highest in young patients affected by active RRMS with low-to-moderate disability [232]. The reduction of brain atrophy rates to values comparable to healthy controls was also reported, usually preceded by a transient increase of brain volume loss, mostly attributed to pseudo-atrophy when using intermediate-intensity regimens [234, 235, 238, 239].

The superior effectiveness of AHSCT with lymphoablative protocol (CY + ATG) compared with available DMTs (alemtuzumab and ocrelizumab excluded) was demonstrated in RRMS by the RCT MIST: the proportion of patients with disease progression at year 3 was 5.19% in the AHSCT group compared with 62.5% in the DMTs group, with an HR of 0.07 (95% CI, 0.02-0.24; *p <* 0.001) [240]. The proportion of cases with relapse at year 3 was 9.61% in the AHSCT group *vs*. 79.63% in the DMTs group. NEDA in the AHSCT and DMTs group was 90.3% *vs*. 5.93% at year 3 and 78.5% *vs.* 2.97% at year 5, respectively. No deaths were reported; the rate of infection per patient per year was 0.19 in the HSCT group and 0.23 in the DMT group. Secondary autoimmune diseases were diagnosed in 5 AHSCT-treated patients (one idiopathic thrombocytopenic purpura (ITP) and four thyroiditis) and one ITP DMTs-treated patient.

More recently, a monocentric cohort of 507 (414 RR, 93 SP) MS patients treated with non-myeloablative AHSCT (CY-based protocol) at Northwestern University between July 2003 and October 2019 confirmed the overall effectiveness of the procedure [241]. In the whole cohort, relapse-free survival was 89.1% at year 5, and progression-free survival was 89.6% at year 4, the latter higher in RRMS (96.8%) compared with SPMS (66%). Furthermore, a significant improvement in EDSS was observed after the transplant, which was sustained up to year 5 in RRMS. Transplant-related mortality was 0.19% (1 death due to hospital-acquired legionella pneumonia of 507 treated). Two deaths due to neoplasms were reported: one colon cancer at year 3 and one T cell lymphoma at year 10 (this latter patient had received alemtuzumab in the conditioning regimen). As secondary autoimmune diseases, 10 cases of ITP occurred (mostly in patients treated with alemtuzumab-containing regimens), and roughly 10% of the cases developed autoimmune thyroiditis.

On grounds of efficacy and increasing safety profile, AHSCT has been endorsed by the EBMT and the American Society for Blood and Marrow Transplantation as a standard of care for RRMS refractory to conventional DMTs [221, 242]. Comparative prospective studies further exploring this topic are ongoing.

#### 5.15.2 Cyclophosphamide (CY, Endoxan Baxter^®^)

Cyclophosphamide (CY) is an alkylating drug with an immunosuppressive effect, chemically related to nitrogen mustards, that can be administered orally or, more efficaciously, intravenously with different dosing regimens. CY is transformed in the liver into active metabolites, which interfere with the replication of rapidly proliferating cells, including T cells and B cells, inducing a suppression of cell-mediated and humoral immunity [243]. In MS, a reduction of pro-inflammatory Th1 cytokines interferon-γ and IL-12, increased secretion of the anti-inflammatory Th2 cytokines IL-4 and IL-10 in CSF and peripheral blood, and a re-shaping of T cell subsets towards a less inflammatory phenotype were reported [244]. CY crosses the BBB in a dose-dependent manner, and therefore, the blood-CSF ratio for active metabolites of this molecule is remarkably improved by IV administration [245]. In addition, a possible activation of the drug within the CNS was suggested [246, 247]. These data indicate that at least part of the CY effect is also exerted on chronic inflammation compartmentalized in the CNS, making this medication particularly suitable for the treatment of patients with PIRA phenotype (Table **1**).

CY is widely used for the treatment of malignant diseases, but therapeutic indications include several autoimmune diseases, including neurological disorders; in Italy, it is approved for the treatment of “autoimmune diseases of the nervous system” [248]. The first use of CY in MS dates back to 1996 [249]. The effectiveness of CY on relapse activity was observed both in RRMS and SPMS [250-253]. The effectiveness of CY in SPMS was also suggested by an open-labelled head-to-head study in SPMS, showing an effect on both relapses and disability similar to mitoxantrone [254]. This latter treatment proved to be superior to a placebo in an RCT in SPMS [255]. Finally, CY is considered a therapeutic option in countries with limited access to high-efficacy therapies [256].

ADRs include alopecia, nausea/vomiting, transient myelosuppression, haemorrhagic cystitis, amenorrhea, and transient azoospermia. Long-term adverse events include risk for secondary malignancies (mainly bladder cancer) and secondary infertility. CY is contraindicated in case of hypersensitivity, urinary outflow obstruction, active infections, cystitis, and severe bone marrow impairment. It cannot be administered during pregnancy and breastfeeding.

## 6 TREATMENT PARADIGMS: INDUCTION, ESCALATION, AND SEQUENCING

In light of the plurality of DMTs currently available, which differs in terms of mechanism of action, efficacy, safety, tolerability, and costs, extensively shared decision-making between both clinicians and patients is required for the choice of the most appropriate medication. Treatment decisions should be guided by the clinical and demographic characteristics of the individual patient and include assessment of prognostic factors in view of a “tailored” treatment plan. Indeed, the foundations of personalized medicine lie in the concept of “prognostication” *i.e*., the prediction of a plausible course considering several aspects such as demographics and environmental factors, clinical features, MRI measures, and biomarkers. Moreover, as the disease features often change during its course, rigorous clinical and MRI monitoring is required to perform any adjustments in therapy when necessary [257]. The availability of DMTs approved for the treatment of progressive MS also requires periodic re-assessments of disease course in order to timely diagnose the transition to SPMS and offer the patient the most appropriate treatment.

It is worth pointing out that in 2018, the first guidelines for MS treatment from the European Committee for Treatment and Research in Multiple Sclerosis (ECTRIMS) have been published [258]. The expert panel has drawn up 21 recommendations covering treatment efficacy, response criteria, strategies to address suboptimal response and safety concerns.

Both the ECTRIMS and the American Academy of Neurology (AAN) guidelines [259] emphasize the importance of earlier initiation and treatment escalation if suboptimal response is observed. Nonetheless, the guidelines do not indicate which DMTs are best to use or how to perform treatment sequencing, leading to poor consensus among physicians on the finest approach to tackle MS, with special regard to the early stages of the disease.

Clinicians may exert the option to adopt two main treatment strategies: escalation and early intensive treatment. The escalation treatment paradigm consists of using, initially, safer but less potent first-line therapies, then upscaling to higher-efficacy DMTs when the patient’s response becomes inadequate. Conversely, the early intensive treatment schedule involves short-term administration of high-efficacy drugs as induction, followed by a maintenance immunomodulating therapy. The success of the latter regimen depends upon administering immunosuppressive drugs for the minimum time required to achieve adequate control over disease activity, followed by a safer maintenance treatment [260]. The adoption of the traditional paradigm of escalation can be supported by the fact that first-line drugs are better known in terms of long-term safety, whereas the recent introduction of highly effective therapies entails the possible occurrence of yet unknown effects of chronic intake. Additionally, high-efficacy medications, *per se*, may expose the patient to substantial risk in the long run. Notwithstanding, a growing body of evidence suggests that the abovementioned risks are less likely to occur in younger patients, who may mostly benefit from the early use of high-efficacy DMTs [261].

Noteworthy, based on the main mechanism by which they interfere with pathophysiological features, currently available medications can be classified into three major groups: predominantly immunomodulating, anti-trafficking, and immune cell-depleting agents. The different mechanisms of action should be considered in the framework of sequencing, bearing in mind that some of those might be complementary, whereas others might contribute to potential toxicity [262].

To date, the escalation strategy is the most commonly used treatment algorithm for early naive MS patients, with the exception of individuals with poor prognostic factors at baseline, candidates for high-efficacy DMTs at the outset.

However, as the therapeutic armamentarium has expanded, clinicians have become increasingly interested in the perspective of initiating aggressive treatment at the time of diagnosis. This approach is based on the hypothesis that it may increase the likelihood of preventing disability progression over time. In fact, individuals who initiate the treatment later in the course of MS do not reach the same goals as those who begin therapy at early stages [263].

Notably, the advantages of early initiation treatment with DMTs are gradually emerging from real-world cohort studies [264], systematic reviews, and meta-analyses [265], consistently with less recent observational studies performed to show related benefits [266]. The common objective of such studies is to establish which approach to use and when by comparing long-term outcomes, safety, and costs.

In particular, two pivotal clinical trials comparing both therapeutic approaches are still recruiting participants. The first one is designed as an interventional, randomized, parallel assignment, blinded treatment study entitled “A Pragmatic Trial to Evaluate the Intermediate-term Effects of Early, Aggressive *Versus* Escalation Therapy in People With Multiple Sclerosis” or “Traditional *Versus* Early Aggressive Therapy for Multiple Sclerosis Trial” (TREAT-MS) (NCT03500^328^]. Its primary endpoint is represented by the time to sustained disability progression measured by the EDSS. The second study is an interventional, randomized, parallel assignment, open label, phase 4 treatment study, officially named “Determining the Effectiveness of earLy Intensive *Versus* Escalation Approaches for the Treatment of Relapsing-Remitting Multiple Sclerosis” (DELIVER-MS) (NCT03535^298^]. Brain volume loss, baselined to month 36, has been established as primary endpoint. As the trials are still ongoing, no results have yet been published [267].

In summary, the escalation treatment allows the patient to take safer medications while still achieving adequate disease control. However, failure to recognise the threshold at which a sub-optimal response is observed may expose the patient to the accumulation of disability and poorer long-term outcomes, also considering the lack of treatments that can reverse disability [268]. On the other hand, the use of high-efficacy DMTs as first-line treatment is supported by consistent evidence from head-to-head RCTs that have shown their superiority over first-line options on long-term disability outcomes and their better performance at earlier stages of MS [269, 270]. On these grounds, early and unrestricted access to high-efficacy DMTs has recently been advocated [271].

Even though significant progress has been made in MS management, the most appropriate therapeutic algorithm is still a matter of ongoing debate and far from being well-defined. Neurologists should take into account individual medical co-morbidities, disease severity, specific adverse effects, and adherence/accessibility prior to the selection of reasonable alternatives [272]. The choice cannot, of course, ignore the individual preferences and needs of patients.

Given the rapidly evolving therapeutic scenario and different efficacy/safety profiles of available DMTs, further research is needed to implement prospective definitions of aggressive MS and validate prognostic factors that may aid the clinician in tailoring treatment strategies to each individual patient.

### 6.1 Specific Subpopulation Management

Managing MS becomes even more challenging when dealing with specific subpopulations, such as patients with co-morbidities, elderly, and pregnant women. In all cases, a carefully revised patient’s medical history must be collected prior to choosing the most suitable treatment regimen.

As suggested by a large retrospective study, certain co-morbidities may affect the effectiveness and safety of specific DMTs [273]. For instance, a history of cardiovascular disease contraindicates fingolimod [274], as well as a past medical record of progressive multifocal leukoencephalopathy (PML) discourages the use of natalizumab. Therefore, chances of initiating a DMT decline as the number of co-morbidities increases, so their presence inevitably impacts the management strategy. Remarkably, the presence of comorbidities was associated with an increased risk of switching from the first drug due to intolerance rather than influencing the initial choice of DMTs [275].

Clearly, aging brings about a greater possibility of experiencing co-morbidities, which can compromise the therapy's effectiveness and often patient adherence. Thus, the elderly still appear underserved by the currently available treatment plans. Moreover, a distinction between senescence-related co-morbidities and sequelae of MS appears challenging. Compared to their younger counterparts, elderly patients show prominent neurodegeneration and few reparative processes, as well as changes in the immune system consistent with immunosenescence [276], resulting in a different response to immunomodulating agents. Another issue concerns the insufficient knowledge of the efficacy and safety of these drugs in older people due to the systematic exclusion of them from clinical trials of approved DMTs [277].

As co-morbidities are often either preventable or curable, their management should be integrated into MS care programs, thus reducing the global burden of disease and ameliorating long-term outcomes.

MS commonly affects women of childbearing age, making pregnancy a main issue to address, and counselling on family planning is generally performed shortly after MS diagnosis. Clinicians should advise the patients about possible implications prior to conception, as many DMTs are not safe during gestation; therefore, the desire for pregnancy and its timing may also affect treatment strategy. Furthermore, pregnancy itself acts as a disease modifier, with a reported reduction of relapse rate of about 70% in the third trimester, followed by a relative increase in risk of disease reactivation in the postpartum [278]. Women newly diagnosed with CIS or RRMS with favourable prognostic indicators intending to become pregnant within a short period might be initiated on DMTs after child conception. Conversely, patients on injectable therapy can continue their treatment while attempting to conceive, with the aim of reducing the risk of experiencing breakthrough disease activity [257].

Different strategies for pregnancy planning may be explored if negative prognostic indicators or highly active MS occur. The use of induction agents like alemtuzumab could potentially enhance the probability of maintaining disease remission during pregnancy and breastfeeding, although this hypothesis remains unproven. Nevertheless, this approach necessitates meticulous planning to guarantee a washout of ≥ 4-6 months between the last course of therapy and conception [187].

Prospective studies suggest that glatiramer acetate and IFNβ are safe during the first trimester of pregnancy and do not raise the risk of miscarriage or fetal malformations [279].

A multidisciplinary team involving neurologists, maternal and fetal specialists, and lactation experts may work closely in planning the most appropriate therapeutic regimen and evaluating the risk/benefit ratio for each DMTs, for both the mother and the child [280].

Specific contraindications for each medical product are accurately discussed in the previous paragraphs, as well as affections discouraging the drug administration.

### 6.2 Focus on the Effects of DMTs on Chronic Active Lesions and Atrophy

Chronic active lesions may be detected by MRI over longitudinal follow-up as slowly expanding lesions (SELs) that are characterized by progressive enlargement, lower T1 intensity at baseline (suggestive of axonal loss) and a larger decrease in T1 intensity over time compared to non-expanding lesions [281].

Iron-enriched macrophages and microglia are located at the edges of chronic active lesions (and, to a minor extent, of chronic inactive lesions), allowing the identification of some smouldering lesions as paramagnetic rim positive lesions (PRLs) [282] using susceptibility-weighted imaging (SWI) [283] and quantitative susceptibility mapping (QSM) [284]. As SELs and PRLs are associated with greater disability and serum/MRI markers of tissue damage [285-287], they have been suggested as potential biomarkers of smouldering inflammation and outcome measures of treatment response.

In PPMS patients participating in the ORATORIO study, ocrelizumab reduced the relative volume of SELs and T1-weighted *in vivo* measures of chronic lesion activity in SELs and in non-SEL areas of pre-existing lesions compared to placebo, although the overall prevalence of SELs was not impacted greatly by the treatment [288]. In SPMS patients from the ASCEND phase III clinical trial, natalizumab reduced SEL prevalence and chronic lesion activity (defined as change over time in T1 lesion volume) in SELs and non-SELs areas compared to placebo [289].

PRLs may appear over time despite treatment with DMTs, but their number is stable in most cases at follow-up. Although the iron rim itself tends to persist, it gradually wanes over several years and may eventually disappear [290]. The reduction over time of average susceptibility within PRL [291] using QSM may, therefore, represent a more dynamic biomarker than PRL number.

SELs and PRLs were recently selected as exploratory endpoints in the phase II trial of tolebrutinib: PRL number was stable at follow-up in 14/16 cases with PRL at baseline (out of 32 evaluable cases), with the disappearance of one PRL in the remaining two [292]. In a small cohort, a greater reduction in QSM within PRL was reported with the use of dimethyl-fumarate compared to glatiramer-acetate [293].

Data available so far on longitudinal changes in SELs and PRLs under treatment with DMTs overall suggest that the effect of high-efficacy DMTs on SELs seems to be modest [294] when compared to the great impact they exert on new/gadolinium-enhancing lesions. Further research is needed to assess whether SELs and PRLs may represent biomarkers of smouldering inflammation and predict treatment response in terms of PIRA.

Brain volume loss (BVL) is accelerated in MS patients compared to healthy people, reflects ongoing axonal damage, and correlates with disability accrual [295, 296]. The effect of DMTs on brain atrophy was correlated with the effect on disability progression in RRMS [297], and BVL is currently adopted as an outcome measure in most RCTs. DMTs variably affect rates of BVL, being the reduction generally greatest for high-efficacy DMTs compared to low-efficacy DMTs [298].

Besides BVL, spinal cord atrophy strongly predicts disability over follow-up [299, 300], but it has been scarcely used as an outcome in clinical trials, and when reported, results were negative [301]. Nonetheless, implementation of spinal cord assessment in the research setting and clinical practice is warranted due to its high informative potential.

## 7 EMERGING DISEASE-MODIFYING THERAPIES

### 7.1 Ublituximab (TG-1101)

Ublituximab is a novel, type I chimeric, immunoglobulin G1 (IgG1) anti-CD20 mAb that binds to an epitope on CD20 that is distinct from the epitopes targeted by other anti-CD20 antibodies [302, 303]. Ublituximab is glycoengineered with a low fucose content in its fragment crystallizable (Fc) region to enhance affinity for all variants of FcγRIIIa (or CD16a) receptors and activating NK cell function, thereby producing potent ADCC while maintaining complement-mediated lysis. Originally, this molecule was developed for the treatment of non-Hodgkin's lymphoma and chronic lymphatic leukaemia, later it was deemed useful to study it also in autoimmune diseases such as MS, systemic lupus erythematosus, and rheumatoid arthritis [304, 305]. Indeed, mAb targeting the B cell antigen CD20 (ocrelizumab and ofatumumab) has demonstrated efficacy in patients with multiple sclerosis [306].

Two identical, phase 3, randomized, multicenter, double-blinded, active-controlled studies (UbLiTuximab In Multiple Sclerosis Treatment Effects - ULTIMATE I and II) enrolled 1.094 participants with RRMS to receive intravenous ublituximab (150 mg on day 1, followed by 450 mg on day 15 and at weeks 24, 48, and 72) and oral placebo or oral teriflunomide (14 mg once daily) and intravenous placebo. The primary endpoint was the annualized relapse rate. Secondary endpoints included the number of gadolinium-enhancing lesions on MRI by 96 weeks and worsening of disability (NCT03277261; NCT03277248) [307, 308].

These studies showed that in patients with RRMS, ublituximab resulted in lower annualized relapse rates and fewer brain lesions on MRI than teriflunomide over a period of 2 years, but did not result in a significantly lower risk of worsening disability [306].

The most commonly reported adverse events that occurred in at least 10% of ublituximab recipients were infusion-related reactions (47.7%), headache (34.3%), nasopharyngitis (18.3%), pyrexia (13.9%), and nausea (10.6%) [306].

### 7.2 Bruton’s tyrosine kinase inhibitors (BTKi)

BTK is a 659 amino acid protein that contains five signalling domains, which is characteristic for members of the Tec family, expressed in most hematopoietic cells, especially B cells, myeloid cells, and platelets, whereas T lymphocytes and plasma cells have low or undetectable levels of BTK. It is a crucial signalling element in B lymphocytes and myeloid cells, including peripheral monocytes or macrophages and CNS-resident microglia. Thus, inhibition of BTK was hypothesised to reduce the acute inflammation associated with contrast-enhancing lesions by modulating B lymphocytes [292, 309, 310].

#### 7.2.1 Evobrutinib (M2951)

Evobrutinib is an oral selective BTKi that blocks B cell activation, cytokines release and has been shown to inhibit the activation, differentiation, and polarisation of proinflammatory M1 macrophages [311] and their cytokines release *in vitro* and has shown *in vivo* efficacy against experimental autoimmune encephalomyelitis [312].

BTKi are currently under investigation in several types of autoimmune diseases because this kinase transmits signals through a variety of receptors in B cells and myeloid cells, so it represents a rational target [313-315].

Montalban *et al.* published the results of phase 2, randomized, double-blind, placebo-controlled study with a parallel, open-label, active control group (dimethyl fumarate), which aimed to evaluate the safety and effectiveness of evobrutinib in 267 participants with RRMS. Participants were placed into 1 of 3 groups to receive evobrutinib (at a dose of 25 mg once daily, 75 mg once daily, or 75 mg twice daily), placebo or dimethyl fumarate (at a dose of 120 mg twice daily for the first week and 240 mg twice daily thereafter) for 24 weeks. After week 24, the participants in the placebo group were switched to receive evobrutinib (25 mg orally administrated once daily from week 24 to week 48), whereas patients who were receiving evobrutinib or DMF continued to be given the same dose. The primary endpoint was the total number of gadolinium-enhancing lesions identified on T1-weighted MRI at weeks 12, 16, 20, and 24. Secondary endpoints were ARR at week 24, Qualified Relapse-Free Status at week 24, change from baseline in EDSS at week 24, and safety (NCT02975349) [316].

The study results showed that patients affected by RRMS who received 75 mg of evobrutinib once daily had significantly fewer enhancing lesions during weeks 12 through 24 than those who received placebo. There was no significant difference with placebo for either the 25 mg once daily or 75 mg twice daily dose of evobrutinib, nor in the annualized relapse rate or disability progression at any dosage. The most commonly observed adverse events associated with evobrutinib were nasopharyngitis and increases in levels of alanine aminotransferase (ALT), aspartate aminotransferase (AST), and lipase [312].

Based on the results of the phase 2 study, two identical phase 3 multicentre, randomized, parallel-group, double-blind, double-dummy trials evaluating the efficacy and safety of evobrutinib administered orally twice daily *versus* interferon β-1a (Avonex^®^) intramuscularly administered once a week in participants with RRMS have been initiated, but they were both terminated (NCT04032158; NCT04032171) [317, 318].

#### 7.2.2 Tolebrutinib (SAR442168)

Tolebrutinib is a small molecule orally administrated that irreversibly binds to BTK, inhibiting it. Reich *et al.* published the results of a phase 2b, randomized, double-blind, placebo-controlled, crossover, dose-finding trial, which aimed to evaluate, as a primary objective, the dose-response relationship for tolebrutinib to reduce the number of new active brain lesions detected using MRI in 130 participants with RRMS and PPMS. The secondary objectives were to evaluate the safety, tolerability, and efficacy of tolebrutinib. After recruitment, participants were assigned to one of two cohorts. Subsequently, they were randomly assigned to one of four tolebrutinib dose groups (5 mg, 15 mg, 30 mg, or 60 mg), giving a total of eight treatment groups. The total study duration was 24 weeks, which included a screening period of 4 weeks and a treatment period of 16 weeks, during which cohort 1 received tolebrutinib (orally administrated once daily) for 12 weeks, then matched placebo (identical-looking tablets) for 4 weeks; cohort 2 received 4 weeks of placebo followed by 12 weeks of tolebrutinib. The last 4 weeks were intended for the patient’s follow-up (NCT03889639) [319].

The study results demonstrated that tolebrutinib treatment led to a dose-dependent reduction in new gadolinium-enhancing lesions, the 60 mg dose being the most efficacious, and the drug was well tolerated. Moreover, the reduction of acute inflammation, combined with the potential to modulate the immune response within the CNS, provides a scientific rationale for pursuing phase 3 clinical trials of tolebrutinib [292].

The results of the phase 2b study led to the start of four phase 3 trials, named GEMINI 1-2 (Relapsing Forms of Multiple Sclerosis Study of Bruton's Tyrosine Kinase Inhibitor Tolebrutinib), PERSEUS (Primary Progressive Multiple Sclerosis Study of Bruton's Tyrosine Kinase Inhibitor Tolebrutinib), and HERCULES (Nonrelapsing Secondary Progressive Multiple Sclerosis Study of Bruton's Tyrosine Kinase Inhibitor Tolebrutinib).

GEMINI 1 and 2 will evaluate the efficacy and safety of tolebrutinib on relapse rates in people with RRMS. Participants, about 900 in each of the two studies, will be randomly assigned to receive tolebrutinib or teriflunomide in a 1:1 ratio. This means that half of the participants will take tolebrutinib and half teriflunomide. The primary objective of these studies is to compare the average number of relapses per year between the two treatment groups. Further objectives include the assessment of worsening or improvement of disability as measured by EDSS, disease activity on brain MRI, and the safety and tolerability of the drug (NCT04410978; NCT04410991) [320, 321].

PERSEUS study will evaluate the efficacy and safety of tolebrutinib in delaying disability progression in people with PPMS. Approximately 990 participants will be randomly assigned to receive tolebrutinib or placebo in a 2:1 ratio. This means that two-thirds of the participants will take tolebrutinib and the other third a placebo. The primary objective of this study will be to compare the progression of disability as defined by the EDSS score. Further objectives include, for instance, the assessment of lesions visible on MRI, as well as the safety and tolerability of the study drug (NCT04458051) [322].

HERCULES study will evaluate the efficacy and safety of tolebrutinib in delaying the progression of disability in people with secondary progressive MS in the absence of relapse (non-relapse) (rfSPMS). 1,290 participants will be randomly assigned to receive tolebrutinib or placebo in a 2:1 ratio. This means that two-thirds of the participants will take tolebrutinib and the other third a placebo. The primary objective of this study will be to compare the time to onset of disability progression, as defined by EDSS, persisting for six months between the 2 treatment groups. Further objectives include, for instance, the assessment of visible lesions on MRI scans, as well as the safety and tolerability of the study drug (NCT04411641) [323].

These trials are all active, but none of them have yet begun patient recruitment.

#### 7.2.3 Fenebrutinib

Fenebrutinib is another member of the BTKi, reported to have the highest potency when compared to the other oral agents evobrutinib and tolebrutinib. It has also previously been proven to be more highly selective than evobrutinib or tolebrutinib [324].

Currently, three phase 3 trials are ongoing, named FENtrepid (A Study to Evaluate the Efficacy and Safety of Fenebrutinib Compared With Ocrelizumab In Adult Participants With Primary Progressive Multiple Sclerosis), and FENhance 1-2 (Study to Evaluate the Efficacy and Safety of Fenebrutinib Compared With Teriflunomide In Relapsing Multiple Sclerosis).

FENtrepid is a multicentre, randomized, double-blind, double-dummy, parallel-group study with the aim of evaluating the efficacy and safety of fenebrutinib on disability progression in adult participants with PPMS. All eligible participants will be randomized 1:1 to either daily oral fenebrutinib (or placebo) or intravenous ocrelizumab (or placebo) in a blinded way through an interactive voice or web-based response system (IxRS). 946 participants will be enrolled and will be recruited globally. Participants who discontinue study medication early or discontinue from the study will not be replaced (NCT04544449) [325].

FENhance 1 and 2 are two multicentre randomized, double-blind, double-dummy, parallel-group studies aimed to evaluate the efficacy and safety of fenebrutinib on disability progression and relapse rate in adult participants with RRMS. Participants, about 736 in each of the two studies, will be randomly assigned to receive fenebrutinib or teriflunomide in a 1:1 ratio (NCT04586023; NCT04586010) [326, 327].

At the present time, all of these trials are still recruiting participants, so the results are not available.

Oh *et al.* reported, in their review of safety data, that the most common AEs associated with drug administration are nasopharyngitis (6%), nausea (5.7%), and headache (5.4%) [328].

#### 7.2.4 Orelabrutinib

Orelabrutinib is a second-generation, orally administered, potent, irreversible, and highly selective BTKi being developed for the treatment of B cell malignancies and autoimmune diseases. In December 2020, orelabrutinib was first approved in China for the treatment of patients with mantle cell lymphoma (MCL) or chronic lymphocytic leukaemia (CLL)/small lymphocytic lymphoma (SLL), who have received at least one previous treatment [329].

Currently, is ongoing a phase 2 randomized, double-blind, placebo-controlled trial has been performed in patients with RRMS to evaluate the efficacy, safety, tolerability, pharmacokinetics, and biological activity of different doses of orelabrutinib. The study will enrol approximately 160 participants who will be assigned randomly to 1 of 4 treatment groups; placebo, low dose orelabrutinib, medium dose orelabrutinib, or high dose orelabrutinib at a 1:1:1:1 ratio for a period of 12 weeks. The primary outcome being measured in this study is the cumulative number of gd-enhancing T1 MRI brain lesions. Investigators are still recruiting participants, so preliminary data are not available (NCT04711148) [330].

### 7.3 Masitinib (AB1010)

Masitinib is a selective tyrosine kinase inhibitor particularly efficient in controlling the survival, migration, and degranulation of mast cells through the inhibition of essential growth and activation signalling pathways [331].

The first clinical trial aiming to evaluate the safety and efficacy of masitinib in MS was a phase 2a, randomized, double-blind, placebo-controlled study performed by Vermersch *et al.* This study enrolled 35 participants with PPMS or relapse-free secondary progressive multiple sclerosis (rfSPMS) who received oral masitinib administered at 2 dose levels for three years. Patients were randomized to groups of masitinib treatment of 3-6 mg/kg/day or placebo, and the primary endpoint was the level of improvement in multiple sclerosis functional composite (MSFC) scores (NCT01450488) [332]. Response data at 12 months showed an increase in MSFC score in patients included in the masitinib treatment group *versus* a decrease of the same score in the placebo group. Notwithstanding, the EDSS remained stable for both treatment groups. This difference between masitinib and placebo persisted until the end of the study at 18 months [333].

Although the results were not statistically significant, they gave reason to begin a phase 3 study with the purpose of comparing the safety and effectiveness of masitinib *versus* placebo in the treatment of patients with PPMS or rfSPMS. The trial enrolled 656 subjects, who received either masitinib 4.5 mg/kg/day orally twice daily, masitinib 4.5 mg/kg/day orally twice daily with dose escalation to 6 mg/kg/day after 3 months of treatment, placebo orally twice daily, or placebo orally twice daily with a matched dose escalation after 3 months of treatment. The primary outcome was EDSS score after 96 weeks of treatment; secondary outcomes included MS quality of life 54 items (MSQOL-54) and MSFC score (NCT01433497) [334]. Despite the completion of the study in 2020, the findings have yet to be published.

Concerning adverse events, the phase 2a trial highlighted that the most frequent of them were mild to moderate and transitory, including asthenia (41%), rash (26%), nausea (22%), edema (19%), and diarrhea (11%) [333].

### 7.4 Ibudilast (MN-166)

Ibudilast is a small molecule available in Asia for the treatment of asthma and poststroke vertigo. Ibudilast inhibits several cyclic nucleotide phosphodiesterases, macrophage migration inhibitory factor, and toll-like receptor 4 and can cross the BBB, potentially having effects in the CNS [335, 336]. Levels of macrophage migration inhibitory factor and toll-like receptor 4 are increased in the cerebrospinal fluid (CSF) of patients with progressive multiple sclerosis, and these proteins can elicit inflammatory responses in the CNS [337, 338].

The first phase 2 clinical study aimed to evaluate the efficacy, tolerability, and effects on MRI parameters of two different doses of ibudilast was performed by Barkhof *et al.* in subjects affected from RRMS. This was a multicentre, double-blind trial that enrolled 297 patients randomly assigned to receive 30 or 60 mg ibudilast or placebo every day for 12 months. The primary endpoint was the cumulative number of newly active lesions on bimonthly brain MRI over 12 months. Secondary endpoints included relapse rate, changes in EDSS, T2-hyperintense and T1-hypointense lesion volumes, and percent brain volume change (PBVC). At the end of the study, the authors concluded that ibudilast showed no beneficial effects on the rate of newly active lesions and relapses. However, preliminary evidence suggested that ibudilast seemed to act in a neuroprotective manner as measured by 2 independent MRI outcomes, with a possible beneficial clinical effect on disability progression [339].

A second phase 2 trial, concluded in July 2020, was conducted to evaluate ibudilast safety, tolerability, and activity in patients with PPMS and SPMS. This was a randomized, double-blind, placebo-controlled study that included 255 subjects to receive oral ibudilast (≤ 100 mg daily) or placebo for 96 weeks. The primary endpoint was the rate of brain atrophy, as measured by the brain parenchymal fraction (brain size relative to the volume of the outer surface contour of the brain). Secondary endpoints included the change in the pyramidal tracts on diffusion tensor imaging, the magnetization transfer ratio in normal-appearing brain tissue, the thickness of the retinal nerve fiber layer, and cortical atrophy (NCT01982942) [340].

Analysis of study data underlined that ibudilast was associated with slower progression of brain atrophy than placebo but was associated with higher rates of gastrointestinal side effects, headache, and depression that occurred in 92% of patients [341].

### 7.5 Opicinumab (BIIB033)

Opicinumab (or anti-LINGO-1 Li81 mAb) has been the first anti-LINGO-1 monoclonal antibody to enter clinical development. It is a fully human IgG1 aglycosylated monoclonal antibody that binds human LINGO-1 with high affinity and specificity. Moreover, BIIB033 has been engineered to have reduced Fcγ and complement effector functions [342].

LINGO-1 (leucine-rich repeat and Ig-containing Nogo receptor-interacting protein-1), also known as LERN1 and LRRN6A, is a 581 amino acid cell-surface glycoprotein selectively expressed by oligodendrocytes and neurons in the CNS. LINGO-1 expression regulates the timing of CNS myelination during development, inhibiting oligodendrocyte differentiation, myelination, neuronal survival, and axonal regeneration by activating ras homolog gene family member A (RhoA) and inhibiting protein kinase B (Akt) phosphorylation signalling pathways. The glycoprotein expression is upregulated in diverse CNS neuropathologies, including Parkinson’s disease and MS [342-344].

Two phase 2 clinical trials were performed with opicinumab, named SYNERGY and AFFINITY.

SYNERGY is a randomized, double-blind, placebo-controlled, parallel-group, dose-ranging study to assess the efficacy, safety, tolerability, and pharmacokinetics of BIIB033 in 419 subjects with relapsing multiple sclerosis (RRMS and SPMS with relapses) when used concurrently with Avonex^®^.

Participants were randomised to receive opicinumab 3 mg/kg, 10 mg/kg, 30 mg/kg, or 100 mg/kg, or placebo intravenously administered once every 4 weeks for a total period of 72 weeks. Furthermore, all patients self-administered intramuscular interferon-β1a, as background anti-inflammatory treatment once a week up to week 84 (NCT01864148) [345].

Investigators concluded that primary data analysis did not demonstrate a significant dose-linear improvement in disability compared with placebo in patients with relapsing multiple sclerosis [346].

SYNERGY study results paved the way for the second phase 2 trial AFFINITY, which is a multicentre, randomized, double-blind, placebo-controlled study aiming to evaluate the safety and efficacy of opicinumab, as add-on therapy to DMTs, *versus* placebo on disability improvement over 72 weeks in 263 patients with relapsing MS. The secondary objective was to evaluate the mAb effects *versus* placebo on additional measures of disability improvement.

Participants were randomized to receive BIIB033 750 mg intravenously as an add-on therapy to a background DMT once every 4 weeks over 72 weeks in part 1 and once every 4 weeks over 96 weeks in part 2, or placebo intravenously as an add-on therapy to a background DMT once every 4 weeks over 72 weeks in part 1 and opicinumab once every 4 weeks over 96 weeks in part 2 (NCT03222973) [347].

### 7.6 BIIB061

BIIB061 is an oral small molecule with a unique mechanism of action that may provide a pharmacological intervention to overcome the failure of remyelination in all forms of multiple sclerosis by blocking mechanisms that prevent differentiation of oligodendrocytes progenitors.

In September 2019 started a multicentre, double-blind, placebo-controlled, parallel-group, dose-ranging phase 2 study to evaluate the efficacy and safety of oral BIIB061 as add-on therapy to interferon-β1a or glatiramer acetate in approximately 300 patients with RMS.

The primary objectives are to evaluate the safety and efficacy of BIIB061 *versus* placebo in improving disability outcome. The secondary objectives are to assess the effects of BIIB061 *versus* placebo on brain MRI markers of remyelination and axons preservation in chronic multiple sclerosis lesions and to evaluate the effects of BIIB061 *versus* placebo on additional measures of improved disability outcome.

The findings have not been published as the patient recruitment phase has not yet begun (NCT04079088) [348].

Please find below the Fig. (**2**) that summarize the mechanisms of action of approved and under investigation medications and Table **2** that summarize emerging disease-modifying therapies and related clinical trials.

## 8 STEM CELL-BASED APPROACHES FOR MS

CNS typically has a poor ability to repair and regenerate new neurons because of its limited pool of precursor cells, expression of myelin-associated growth inhibitory factors, and the inherent propensity of resident glial cells to form scar tissue. At present, it is very difficult to treat CNS diseases with conventional clinical therapies. Therefore, some studies have suggested that stem cell treatment may offer a novel therapeutic strategy for CNS disease [349].

Among stem cells, mesenchymal stem cells (MSCs) have been considered the best source that overcome limitations related to ethical issues, tumorigenic activity and probable rejection of transplanted cells [350]. They are multipotent stem cells with the neurogenic potential that make them good candidates for different types of nervous tissues [351, 352].

MSCs harvested from adult tissues have been described as important therapeutic cell sources for the treatment of CNS and PNS perturbations since they possess the capacity for both neuronal and glial differentiation. They are characterized by highly proliferative activity, are capable of self-renewal, and have immunomodulatory and neuroregenerative effects, as well as express numerous anti-inflammatory and neurotrophic factors supporting nerve repair [353]. MSCs can be easily isolated from peripheral blood, bone marrow, adipose tissue, umbilical cord (UC), and placenta, and have recently provided a ray of hope for treating MS patients since it seems that upon intravenous injection into the cerebrospinal fluid, these cells are able to locate into brain lesions [354].

The main MSCs used so far for clinical trials in MS include bone marrow-derived MSCs, human adipose-derived MSCs and umbilical cord MSCs. A brief focus will also be dedicated to human dental pulp stem cells.

### 8.1 Bone-marrow-derived MSCs

Bone marrow tissue is an important source of MSCs [355]. These cells have the ability to differentiate into several cell types due to their multi-potential properties. A number of clinical trials have evaluated the effectiveness of autologous BM-MSCs in MS disease treatment [354] (NCT02166021, NCT02403947, NCT00395200, NCT00781872, NCT020-35514, NCT03069170, NCT00014755, NCT02495766). The cells were administered intravenously, improved neurobehavior outcomes and reduced inflammatory infiltration as well as demyelination in the spinal cord. The results were centered on the improvement in disease severity, cognitive function, and quality of life of MS patients, thanks to the cells’ neuroprotective and anti-inflammatory properties [354].

### 8.2 Adipose-derived MSCs

Human adipose-derived MSCs (AD-MSCs) can be easily isolated by liposuction method from adipose tissue, which is abundant in abdominal tissue and hip area. Experimental studies revealed that AD-MSCs can differentiate into myelin-producing cells and compensate for myelin loss in MS disease models [354]. Both the autologous and allogenic models of ASCs have been frequently used in clinical studies, with several of the studies reporting that the injection of these cells is safe without adverse effects (NCT01056471, NCT01730547).

### 8.3 Umbilical Cord MSCs (UCMSCs)

UMSC can be easily derived from umbilical cords discarded after delivery, so they do not cause any ethical controversies, and their collection method is noninvasive, are genome-stable, have lower immunogenicity, has higher expansion ability compared to those from the bone marrow and other adult tissues, and have more powerful immune-modulating properties [354]. UCMSCs can be isolated from different parts of the umbilical cord, such as Wharton's jelly. MSCs derived from this jelly region have high proliferative and therapeutic ability, their administration is of little invasiveness, and they lack significant immunogenicity (low levels of class I and class II human leukocyte antigen), which permits allogenic transplantation without immunosuppressive drugs. Mainly allogenic UCMSC have been evaluated in clinical trials (NCT03326505, NCT02034188, NCT01364246).

### 8.4 Dental Pulp Stem Cells (DPSCs)

DPSCs are multipotent stem cells that can be considered as a new noninvasive autologous source for MSCs [356]. They can be obtained from the third molars, usually discarded as medical waste. DPSCs have MSC-like characteristics, such as the ability for self-renewal and multilineage differentiation. These dental pulp-derived MSCs prevent ethical concerns as obtained without unnecessary invasive procedures, unlike MSCs collected from bone marrow or adipose tissue [357-359]. DPSCs can secrete neurotrophic factors such as neurotrophin (NT) while differentiating into neuron-like cells [359, 360]. Furthermore, DPSCs express neuron-related markers before being induced to neuronal differentiation [358, 360]. Taken together, these unique properties make DPSCs an excellent candidate for stem cell-related therapies in nerve diseases and attention on DPSCs use in neurodegenerative disease has increased in the last decade (spinal cord injury models, post ischemic neuronal death [361], Parkinson`s disease [362], MPTP induced damage [363], retinal injury [364, 365], optic nerve damage [366, 367]). Because oligodendrocytes are able to produce the myelin sheaths, oligodendrocyte-based cell therapy is suggested as a promising alternative therapy for myelin repair of demyelinated nerves [368]. They show neural characteristics like neurons and can be easily collected from dental tissues [369]. These properties nominate hDPSCs as an appropriate cell source for neuroregenerative medicine. Up to date, the multipotency potential of hDPSCs to generate different lineages, such as osteogenic, adipogenic as well as neurogenic lines, has been investigated [370]. Although differentiation of hDPSCs into neuron cell type has been reported [371], the *in-vitro* oligodendrogenesis potential of these cells and assessment of mature specific markers of differentiated cells have been overlooked. In this regard, previous research studies showed that transplantation of differentiated cells is more effective than engraftment of undifferentiated stem cells [372].

## CONCLUSION

The last four decades, and particularly the last one, have shown a great increase in pharmacological options for the management of multiple sclerosis; a number of these allowed improved therapeutic management as they can actually modify the natural history of the disease. In addition, different lines of treatment are available today, bringing about additional clinical benefits. However, research has focused its attention on identifying new molecular entities with activity in relapsing forms. In fact, as more extensively highlighted above, only a few drugs have today been authorised for the treatment of progressive forms, which indeed appear rather resistant to current therapeutic options. At the same time, the absence of curative therapy, representing the main unmet medical need, as well as the difficulty in MS management, are attributable, at least in part, to its multifactorial etiopathogenesis, characterized by the coexistence of genetic and environmental factors, as well as the adaptive and evolving nature of the immune system, which keeps changing in relation to time and age. Therefore, to date, early intervention following early diagnosis appears to be the most effective approach to control neuroinflammation and neurodegeneration that characterise the progression of MS while using the most effective medication for each patient. Future research into new molecular entities should consider the compelling need to achieve a greater understanding of the immunopathological mechanisms underlying MS so as to identify new targets for therapeutic intervention.

## Figures and Tables

**Fig. (1):**
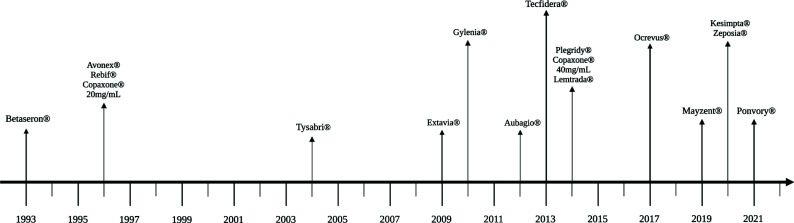
Timeline about FDA licensed disease-modifying treatments. Created with BioRender.com; accessed on 20 April 2023.

**Fig. (2):**
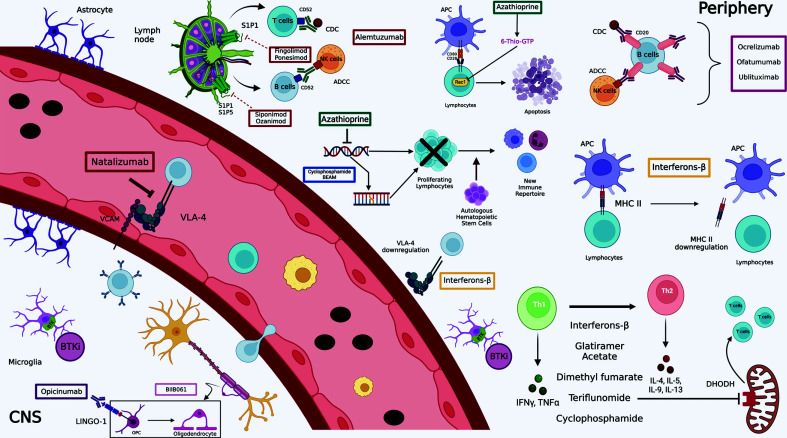
Mechanism of action of approved and under investigation disease-modifying treatments for multiple sclerosis. Abbreviations: CNS, central nervous system; OPC, oligodendrocyte precursor cell; LINGO-1, leucine-rich repeat and Ig containing Nogo receptor-interacting protein-1; BTK, Bruton’s tyrosine kinase; BTKi, Bruton’s tyrosine kinase inhibitors; VLA-4, very late antigen-4; VCAM, vascular cell adhesion molecule; S1P1, sphingosine-1-phosphate 1; S1P5, sphingosine-1-phosphate 5; CDC, complement-dependent cytolysis; ADCC, antibody-dependent cell-mediated cytotoxicity; CD52, Cluster of Differentiation 52; CD28, Cluster of Differentiation 28; CD80, Cluster of Differentiation 80; Rac1, Ras-related C3 botulinum toxin substrate 1; CD20, Cluster of Differentiation 20; NK, Natural Killer; MHC II, major histocompatibility complex II; APC, antigen-presenting cell; DHODH, dihydroorotate dehydrogenase; INF-γ, interferon-γ; TNF-α, tumor necrosis factor-α; BEAM, carmustine, etoposide, cytarabine and melphalan. Created with BioRender.com; accessed on 24 April 2023.

**Table 1:** Current therapeutic strategies for the management of multiple sclerosis.

**Nome of the Drug**	**Pharmaceutical Form**	**Mechanism of Action**	**Adverse Drug Reactions**	**Approval Clinical Trials**	**FDA Approval**
Azathioprine (Jayempi^®^)	10 mg/ml oral suspension.	AZA inhibits purine nucleotide biosynthesis by suppressing DNA and RNA synthesis, thus downregulating B and T cell functions. It can also induce T cell apoptosis through CD28 co-stimulation, mediated by a specific binding of AZA-generated 6-thioguanine triphosphate to Rac1 instead of guanosine-triphosphate.	Bone marrow depression is most frequently expressed as leukopenia and thrombocytopenia, infections, and hypersensitivity.	Not applicable	Off-label
Cyclophosphamide (Endoxan Baxter^®^)	1. 50 mg film-coated tablets. 2. 200 mg powder for solution for injection. 3. 500 mg powder for solution for injection. 4. 1 mg powder for solution for injection.	CY interferes with the replication of T cells and B cells, inducing a suppression of cell-mediated and humoral immunity and a re-shaping of T cell subsets towards a less inflammatory phenotype.	Alopecia, nausea and vomiting, transient myelosuppression, haemorrhagic cystitis, amenorrhea and transient azoospermia, the risk for secondary malignancies, and secondary infertility.	Not applicable	Off-label
Interferons-β1a (Avonex^®^, Rebif^®^) Interferons-β1b (Betaseron^®^/ Betaferon^®^, and Extavia^®^)	Avonex^®^: 30 micrograms/0.5 ml solution for injection pre-filled syringe (for intramuscular use). Rebif^®^: 22 mcg solution for injection in a pre-filled syringe (for subcutaneous and intramuscular use). Betaseron^®^/Betaferon^®^: 250 mcg/ml powder and solvent for solution for injection. Extavia^®^: 250 mcg/ml powder and solvent for solution for injection.	IFN-β exerts a complex mechanism of action down-regulating MHC-class II molecule expression on the antigen-presenting cells (APCs).	Injection site reactions, flu-like symptoms, asthenia, hypersensitivity, myalgia, and liver enzyme elevation.	Avonex^®^: MSCSG Rebif^®^: PRISMS Betaseron^®^/ Betaferon^®^: IFN-β MS Study Group.	Avonex^®^: 1996 Rebif^®^: 1996 Betaseron^®^/ Betaferon^®^: July 1993 Extavia^®^: August 2009
PEGylated IFNβ-1a (Plegridy^®^)	1. 63-94-125 mcg solution for injection in a pre-filled syringe (for subcutaneous use). 2. 63-94-125 mcg solution for injection in a pre-filled pen (for subcutaneous use). 3. 125 mcg solution for injection in a pre-filled syringe (for intramuscular use).	The mechanism of action of peginterferon beta-1a in multiple sclerosis (MS) is not well known. Peginterferon beta-1a binds to the type I interferon receptor on the surface of cells and elicits a cascade of intracellular events leading to the regulation of interferon-responsive gene expression.	General disorders and administration site conditions, gastrointestinal disorders, skin and subcutaneous tissue disorders, musculoskeletal and connective tissue disorders, psychiatric disorders, blood and lymphatic system disorders, and immune system disorders.	Efficacy and Safety Study of Peginterferon Beta-1a in Participants with Relapsing Multiple Sclerosis (ADVANCE).	August 2014
Glatiramer acetate (Copaxone^®^)	1. 20 mg/ml solution for injection in a pre-filled syringe (for intravenous or intramuscular use). 2. 40 mg/ml solution for injection in a pre-filled syringe (for intravenous or intramuscular use).	The exact mechanism of action is not completely understood, but it seems to function as an altered peptide ligand, cross-reacting with the autoantigen MBP, thus promoting regulatory T-cells (T_reg_) instead of stimulating adverse T-cell autoreactivity. The immunomodulatory action of glatiramer acetate probably originates from indiscriminate binding to MHC-II molecules on APCs, displacing MBP from these binding sites, hence in altered T-cell responses.	Injection-site reactions (tenderness, itching, erythema, or induration), as well as mild and transient hypersensitivity reactions of flushing, chest tightness, dyspnoea, palpitations, and anxiety.	Copaxone^®^ 20 mg/ml: Phase III Randomized, Double-Blind, Placebo-Controlled Study of Copolymer 1 for Relapsing-Remitting Multiple Sclerosis. Copaxone^®^ 40 mg/ml: A Study in Subjects with Relapsing-Remitting Multiple Sclerosis (RRMS) to Assess the Efficacy, Safety and Tolerability of GA Injection 40 mg Administered Three Times a Week Compared to Placebo (GALA).	Copaxone^®^ 20 mg/ml: December 1996 Copaxone^®^ 40 mg/ml: January 2014
Teriflunomide (Aubagio^®^)	1. 7 mg film-coated tablets. 2. 14 mg film-coated tablets.	It is the active metabolite of leflunomide, acting as an immunosuppressor *via* the interference with de-novo synthesis of pyrimidine. It selectively and reversibly inhibits the mitochondrial enzyme dihydroorotate dehydrogenase (DHODH), determining a reduction in the proliferation of T-cells assumed to be autoreactive.	Gastrointestinal manifestations, such as diarrhea, abdominal pain, dyspepsia, nausea, and vomiting, increasing liver enzyme levels, susceptibility to infections, hypertension, and hair thinning	Study of Teriflunomide in Reducing the Frequency of Relapses and Accumulation of Disability in Patients with Multiple Sclerosis (TEMSO). An Efficacy Study of Teriflunomide in Participants with Relapsing Multiple Sclerosis (TOWER).	September 2012
Dimethyl fumarate (Tecfidera^®^)	1. 120 mg gastro-resistant hard capsules. 2. 240 mg gastro-resistant hard capsules.	The immunomodulatory effects of dimethyl fumarate (DMF) are exerted through the activation of the nuclear factor (erythroid-derived 2)–like 2 (Nrf-2) pathway and Nrf2-independent pathways.	Nausea, diarrhea, flushing, and abdominal pain.	Efficacy and Safety of Oral BG00012 in Relapsing-Remitting Multiple Sclerosis (DEFINE). Efficacy and Safety Study of Oral BG00012 With Active Reference in Relapsing-Remitting Multiple Sclerosis (CONFIRM).	March 2013
Fingolimod (Gilenya^®^)	1. 0.25 mg hard capsules. 2. 0.5 mg hard capsules.	Acts as a sphingosine-1-phosphate (S1P) receptor antagonist, preventing the egression of lymphocytes from secondary lymphatic tissues and inhibiting entry of autoreactive lymphocytes into the central nervous system. Furthermore, it non-selectively depredates the S1P1 receptors on T cells besides its internalization, reducing their responsiveness to chemotactic signals.	Headache, elevation of liver enzymes, diarrhea, cough, influenza, sinusitis, back pain, bradycardia, and less frequently first or second-degree atrioventricular block for which patients should be monitored for at least 6 hours after the first dose.	Efficacy and Safety of Fingolimod in Patients with Relapsing-remitting Multiple Sclerosis (FREEDOMS). Efficacy and Safety of Fingolimod in Patients with Relapsing-remitting Multiple Sclerosis With Optional Extension Phase (TRANSFORMS).	September 2010
Natalizumab (Tysabri^®^)	1. 300 mg concentrate for solution for infusion. 2. 150 mg solution for injection in a pre-filled syringe.	Humanized recombinant IgG4 monoclonal antibody targeting the α4-integrin molecule, a component of VLA-4, expressed on lymphocytes preventing binding to the ligand vascular cell adhesion molecule (VCAM) found on endothelial cell surfaces. This blocks the adhesion and subsequent extravasation of lymphocytes across the BBB, reducing CNS inflammation.	Injection-site reactions during infusion, increased risk of developing infections, alteration of haematochemical parameters, headache, fatigue, joint pain, vomiting, and hives.	Natalizumab Safety and Efficacy in Relapsing-Remitting Multiple Sclerosis (AFFIRM). Safety and Efficacy of Natalizumab in Combination with Avonex in the Treatment of Multiple Sclerosis SENTINEL).	November 2004
Alemtuzumab (Lemtrada^®^)	12 mg concentrate for solution for infusion.	Humanized monoclonal IgG1-antibody that targets CD52 (molecular weight 21-28 kD), a surface glycoprotein with partially unknown functions predominantly expressed (>95%) on T (CD3^+^) and B (CD19^+^) cells.	Autoimmune-associated diseases, infusion-associated reactions (IARs), infections (especially upper respiratory tract infections and urinary tract infections), heart disease, and lymphoproliferative disorders associated with Epstein-Barr virus. Malignancies such as thyroid cancer, melanoma, and melanoma-*in-situ*, as well as lymphoproliferative disorders, have been reported.	Comparison of Alemtuzumab and Rebif^®^ Efficacy in Multiple Sclerosis, Study One (CARE-MS I). Comparison of Alemtuzumab and Rebif^®^ Efficacy in Multiple Sclerosis, Study Two (CARE-MS II).	November 2014
Rituximab (MabThera^®^)	1. 100 mg concentrate for solution for infusion. 2. 500 mg concentrate for solution for infusion.	It is a chimeric mouse/human IgG1 anti-CD20 monoclonal antibody. The Fab domain of rituximab binds to the CD20 antigen on B lymphocytes, and the Fc domain can recruit immune effector functions to mediate B cell lysis.	Common adverse drug reactions include infections and infestations (such as bacterial infections and viral infections), blood lymphatic system alterations (neutropenia, leucopoenia, anaemia, and thrombocytopenia), immune system disorders (infusion-related reactions), metabolism and nutrition impairments, psychiatric, nervous system, eye, cardiac, and vascular diseases.	Not applicable	Off-label
Ocrelizumab (Ocrevus^®^)	300 mg concentrate for solution for infusion.	Humanised IgG1 anti-CD20 antibody that binds avidly to CD20, a transmembrane phosphoprotein expressed on the surface of mature B cells, leads to a dose-dependent depletion of B cells *via *the ADCC mechanism. Moreover, rituximab is a chimeric antibody and acts predominantly *via *CDC.	Upper respiratory tract infection, nasopharyngitis, influenza, cough, catarrh, blood immunoglobulin M decreased infusion-related reactions, neutropenia.	A Study of Ocrelizumab in Comparison with Interferon Beta-1a (Rebif^®^) in Participants with Relapsing Multiple Sclerosis (OPERA I). A Study of Ocrelizumab in Comparison with Interferon Beta-1a (Rebif^®^) in Participants with Relapsing Multiple Sclerosis (OPERA II).	March 2017
Ofatumumab (Kesimpta^®^)	1. 20 mg solution for injection in a pre-filled syringe. 2. 20 mg solution for injection in a pre-filled pen.	Recombinant fully human anti-CD20 monoclonal immunoglobulin G1 antibody, which binds to a region of the CD20 different from that of other anti-CD20 antibodies. The binding between the FAB portion of ofatumumab and CD20 leads to B cell (and T cell) depletion *via *CDC and ADCC.	Injection-related reactions, nasopharyngitis, headache, injection-site reactions, upper respiratory tract infections, and urinary tract infections.	Efficacy and Safety of Ofatumumab Compared to Teriflunomide in Patients with Relapsing Multiple Sclerosis I and II (ASCLEPIOS I and II).	August 2020
Siponimod (Mayzent^®^)	1. 0.25 mg film-coated tablets. 2. 1 mg film-coated tablets. 3. 2 mg film-coated tablets.	It is an oral selective sphingosine 1-phosphate (S1P) receptor modulator (S1P1 and SIP5). Modulation of S1P1 on peripheral lymphocytes inhibits their egress from lymph nodes and, therefore, infiltration of the CNS. Moreover, siponimod crosses the BBB and preclinical data have shown a reduction of central nervous system inflammation and indicate effects on repair mechanisms *via *modulation of S1P1 on astrocytes and S1P5 on oligodendrocytes.	The most common adverse reactions include headache (15%), hypertension (12.6%), dizziness, lowered heart rate, and increased risk of upper respiratory infections.	A Dose Blinded Extension Study to the CBAF312A2201 Study to Evaluate Long-term Safety, Tolerability and Efficacy of BAF312 Given Orally Once Daily in Patients with Relapsing-remitting Multiple Sclerosis. Exploring the Efficacy and Safety of Siponimod in Patients with Secondary Progressive Multiple Sclerosis (EXPAND).	March 2019
Ozanimod (Zeposia^®^)	1. 0.23 mg hard capsules 2. 0.46 mg hard capsules 3. 0.92 mg hard capsules	It is an oral sphingosine-1-phosphate receptor (S1PR) modulator that selectively targets S1P1 and S1P5 with high affinity, thus preventing circulating autoreactive lymphocytes from entering the CNS from peripheral tissues, as well as reducing their presence in the bloodstream. Ozanimod, acting as a functional antagonist of the aforementioned receptors, determines a sustained internalisation and degradation of S1P1 receptors on lymphocytes, which inhibit their egression from lymph nodes and, as a consequence, their trafficking to inflamed tissue sites.	The most commonly reported adverse reactions (>5%) are nasopharyngitis, alanine aminotransferase (ALT) increased, and gamma-glutamyl transferase increased.	A Phase 2/3, Multi-centre, Randomized, Double-blind, Placebo-controlled (Part A) and Double-blind, Double-dummy, Active-controlled (Part B), Parallel Group Study to Evaluate the Efficacy and Safety of RPC1063 Administered Orally to Relapsing Multiple Sclerosis Patients (RADIANCE). A Phase 3, Multi-Centre, Randomized, Double-Blind, Double-Dummy, Active Controlled, Parallel Group Study to Evaluate the Efficacy and Safety Of RPC1063 Administered Orally to Relapsing Multiple Sclerosis Patients (SUNBEAM).	March 2020
Ponesimod (Ponvory^®^)	1. 2 mg film-coated tablets 2. 3 mg film-coated tablets 3. 4 mg film-coated tablets 4. 5 mg film-coated tablets 5. 6 mg film-coated tablets 6. 7 mg film-coated tablets 7. 8 mg film-coated tablets 8. 9 mg film-coated tablets 9. 10 mg film-coated tablets 10. 20 mg film-coated tablets	It is a selective, orally active, rapidly reversible S1P1 receptor modulator. Ponesimod exerts its immunomodulating activity *via *the functional antagonism of the S1P1 receptor expressed on lymphocytes, thus preventing their egression from lymph nodes and, as a result, their circulation in the blood flow and migration to the sites of inflammation.	The most commonly reported adverse drug reactions are nasopharyngitis (19.7%), alanine aminotransferase increase (17.9%) and upper respiratory tract infection (11%).	Multicentre, Randomized, Double-blind, Parallel-group, Active-controlled, Superiority Study to Compare the Efficacy and Safety of Ponesimod to Teriflunomide in Subjects with Relapsing Multiple Sclerosis (OPTIMUM).	May 2021

**Table 2:** Emerging disease-modifying therapies and related clinical trials.

**Name of the Drug**	**Mechanism of Action**	**Clinical Trials**
Ublituximab (TG-1101)	It is a type I chimeric, immunoglobulin G1 (IgG1) anti-CD20 mAb that binds to an epitope on CD20 that is distinct from the epitopes targeted by other anti-CD20 antibodies.	1. Phase III: UbLiTuximab In Multiple Sclerosis Treatment Effects (ULTIMATE I STUDY). 2. Phase III: UbLiTuximab in Multiple Sclerosis Treatment Effects (ULTIMATE II STUDY).
Evobrutinib (M2951)	It is a selective BTKi that blocks B-cell activation and cytokine release and has been shown to inhibit the activation, differentiation, and polarisation of proinflammatory M1 macrophages.	1. A Randomized, Double-Blind, Placebo-Controlled Phase II Study of M2951 With a Parallel, Open-Label, Active Control Group (Tecfidera) in Patients With Relapsing Multiple Sclerosis to Evaluate Efficacy, Safety, Tolerability, Pharmacokinetics, and Biological Activity. 2. A Phase III, Multicentre, Randomized, Parallel Group, Double-Blind, Double Dummy, Active Controlled Study of Evobrutinib Compared With an Interferon Beta 1a (Avonex^®^), in Participants With Relapsing Multiple Sclerosis to Evaluate Efficacy and Safety. 3. A Phase III, Multicentre, Randomized, Parallel Group, Double-Blind, Double Dummy, Active Controlled Study of Evobrutinib Compared With an Interferon Beta 1a (Avonex^®^), in Participants With RMS to Evaluate Efficacy and Safety.
Tolebrutinib (SAR442168)	It is a small molecule orally administrated that irreversibly binds to BTK, inhibiting it.	1. A Phase 2b Dose-finding Study for SAR442168, a Bruton's Tyrosine Kinase Inhibitor, in Participants With Relapsing Multiple Sclerosis. 2. A Phase 3, Randomized, Double-blind Efficacy and Safety Study Comparing SAR442168 to Teriflunomide (Aubagio^®^) in Participants With Relapsing Forms of Multiple Sclerosis (GEMINI I; GEMINI II). 3. A Phase 3, Randomized, Double-blind, Efficacy and Safety Study Comparing SAR442168 to Placebo in Participants With Primary Progressive Multiple Sclerosis (PERSEUS). 4. A Phase 3, Randomized, Double-blind, Efficacy and Safety Study Comparing SAR442168 to Placebo in Participants With Nonrelapsing Secondary Progressive Multiple Sclerosis (HERCULES).
Fenebrutinib	Member of the BTKi, it is reported to have the highest potency when compared to the other oral agents evobrutinib and tolebrutinib.	1. A Phase III Multicentre, Randomized, Double-Blind, Double-Dummy, Parallel-Group Study To Evaluate The Efficacy And Safety Of Fenebrutinib Compared With Ocrelizumab In Adult Patients With Primary Progressive Multiple Sclerosis (FENtrepid). 2. A Phase III Multicentre Randomized, Double-Blind, Double-Dummy, Parallel-Group Study To Evaluate The Efficacy And Safety Of Fenebrutinib Compared With Teriflunomide In Adult Patients With Relapsing Multiple Sclerosis (FENhance 1; FENhance 2).
Orelabrutinib	Orelabrutinib is second-generation, orally administered, potent, irreversible, and highly selective BTKi.	1. A Randomized, Double-Blind, Placebo-Controlled Phase 2 Study of Orelabrutinib in Patients With Relapsing-Remitting Multiple Sclerosis to Evaluate Efficacy, Safety, Tolerability, Pharmacokinetics, and Biological Activity.
Masitinib (AB1010)	It is a selective tyrosine kinase inhibitor particularly efficient in controlling the survival, migration, and degranulation of mast cells through the inhibition of essential growth and activation signalling pathways.	1. A Phase 2a, Randomized, Double-blind, Placebo-controlled Study to Evaluate the Activity of Oral AB1010 Administered at 2 Dose Levels to Patients With Primary Progressive or Relapse-free Secondary Progressive Multiple Sclerosis. 2. A 96 Week, Prospective, Multicentre, Randomized, Double-blind, Placebo-controlled, 2 Parallel-groups, Phase 3 Study to Compare Efficacy and Safety of Masitinib 4.5 mg/kg/Day *Versus* Placebo in the Treatment of Patients With Primary Progressive or Relapse-free Secondary Progressive Multiple Sclerosis.
Ibudilast (MN-166)	Inhibits several cyclic nucleotide phosphodiesterases, macrophage migration inhibitory factor, and toll-like receptor 4 and can cross the blood-brain barrier (BBB), potentially having effects on the central nervous system.	1. A Phase 2 Randomized, Double-blind, Placebo-controlled Study to Evaluate the Safety, Tolerability and Activity of Ibudilast (MN-166) in Subjects With Progressive Multiple Sclerosis.
Opicinumab (BIIB033)	It is an anti-LINGO-1 monoclonal antibody to enter clinical development. It is a fully human IgG1 aglycosylated monoclonal antibody that binds human LINGO-1 with high affinity and specificity. Moreover, BIIB033 has been engineered to have reduced Fcγ and complement effector functions.	1. A Randomized, Double-Blind, Placebo-Controlled, Parallel-Group, Dose-Ranging Study to Assess the Efficacy, Safety, Tolerability, and Pharmacokinetics of BIIB033 in Subjects With Relapsing Forms of Multiple Sclerosis When Used Concurrently With Avonex ( SYNERGY). 2. A Multicentre, Randomized, Double-Blind, Placebo-Controlled Study With Optional Open-Label Extension in Subjects With Relapsing Multiple Sclerosis to Evaluate the Efficacy and Safety of BIIB033 as an Add-On Therapy to Anti-Inflammatory Disease-Modifying Therapies ( AFFINITY).
BIIB061	It is an oral small molecule with a unique mechanism of action that may provide a pharmacological intervention to overcome the failure of remyelination in all forms of multiple sclerosis by blocking mechanisms that prevent differentiation of oligodendrocytes progenitors.	1. A Multicentre, Double-Blind, Placebo-Controlled, Parallel-Group, Dose-Ranging Phase 2 Study to Evaluate the Efficacy and Safety of Oral BIIB061 as Add-On Therapy to Interferon-Beta 1 or Glatiramer Acetate Therapies in Relapsing Multiple Sclerosis.

## LIST OF ABBREVIATIONS

ADCC: Antibody-dependent Cell-mediated Cytotoxicity
APCs: Antigen-presenting Cells
AV: Atrioventricular
BBB: Blood-brain Barrier
CIS: Clinically Isolated Syndrome
CNS: Central Nervous System
EBV: Epstein-barr Virus
GWAS: Genome-wide Association Studies
HLA: Human Leukocyte Antigen
HO-1: Hemoxygenase-1
IARs: Infusion-associated Reactions
MHC: Major Histocompatibility Complex
MS: Multiple Sclerosis
OLs: Oligodendrocytes
RIS: Radiologically Isolated Syndrome
S1P: Sphingosine-1-phosphate
S1PR: Sphingosine-1-phosphate Receptor
SC: Subcutaneously
